# A Universal System for Highly Efficient Cardiac Differentiation of Human Induced Pluripotent Stem Cells That Eliminates Interline Variability

**DOI:** 10.1371/journal.pone.0018293

**Published:** 2011-04-08

**Authors:** Paul W. Burridge, Susan Thompson, Michal A. Millrod, Seth Weinberg, Xuan Yuan, Ann Peters, Vasiliki Mahairaki, Vassilis E. Koliatsos, Leslie Tung, Elias T. Zambidis

**Affiliations:** 1 Johns Hopkins Institute for Cell Engineering, The Johns Hopkins University School of Medicine, Baltimore, Maryland, United States of America; 2 Department of Biomedical Engineering, The Johns Hopkins University School of Medicine, Baltimore, Maryland, United States of America; 3 Division of Neuropathology, Department of Pathology, The Johns Hopkins University School of Medicine, Baltimore, Maryland, United States of America; 4 Department of Neurology, The Johns Hopkins University School of Medicine, Baltimore, Maryland, United States of America; 5 Department of Psychiatry and Behavioral Sciences, The Johns Hopkins University School of Medicine, Baltimore, Maryland, United States of America; University of Southern California, United States of America

## Abstract

**Background:**

The production of cardiomyocytes from human induced pluripotent stem cells (hiPSC) holds great promise for patient-specific cardiotoxicity drug testing, disease modeling, and cardiac regeneration. However, existing protocols for the differentiation of hiPSC to the cardiac lineage are inefficient and highly variable. We describe a highly efficient system for differentiation of human embryonic stem cells (hESC) and hiPSC to the cardiac lineage. This system eliminated the variability in cardiac differentiation capacity of a variety of human pluripotent stem cells (hPSC), including hiPSC generated from CD34^+^ cord blood using non-viral, non-integrating methods.

**Methodology/Principal Findings:**

We systematically and rigorously optimized >45 experimental variables to develop a universal cardiac differentiation system that produced contracting human embryoid bodies (hEB) with an improved efficiency of 94.7±2.4% in an accelerated nine days from four hESC and seven hiPSC lines tested, including hiPSC derived from neonatal CD34^+^ cord blood and adult fibroblasts using non-integrating episomal plasmids. This cost-effective differentiation method employed forced aggregation hEB formation in a chemically defined medium, along with staged exposure to physiological (5%) oxygen, and optimized concentrations of mesodermal morphogens BMP4 and FGF2, polyvinyl alcohol, serum, and insulin. The contracting hEB derived using these methods were composed of high percentages (64–89%) of cardiac troponin I^+^ cells that displayed ultrastructural properties of functional cardiomyocytes and uniform electrophysiological profiles responsive to cardioactive drugs.

**Conclusion/Significance:**

This efficient and cost-effective universal system for cardiac differentiation of hiPSC allows a potentially unlimited production of functional cardiomyocytes suitable for application to hPSC-based drug development, cardiac disease modeling, and the future generation of clinically-safe nonviral human cardiac cells for regenerative medicine.

## Introduction

Cardiac differentiation of human embryonic stem cells (hESC) and human induced pluripotent stem cells (hiPSC) offers a potentially unlimited source of cardiomyocytes for novel drug discovery and testing, regenerative medicine, and the study of human cardiac development and disease [1]. Cardiac cells differentiated from human pluripotent stem cells (hPSC) display normal cardiac molecular, structural and functional characteristics [2], [3], [4], including the ability to respond physiologically to cardioactive drugs [5]. Although hESC differentiation efficiencies up to 70% (as assessed by the percentage of contracting hEB generated) have been published [3], the most commonly used basic protocol for hESC cardiac differentiation has a low efficiency of ∼8–22% [6], [7], and takes up to 21 days to produce contracting areas. This protocol performs even less efficiently for hiPSC (∼1–25%) and take up to 30 days to generate contracting hEB [8], [9].

Multiple approaches have been described for directed and efficient cardiac differentiation of hESC. These methods include co-culture with END2 (mouse visceral endoderm-like cell) stromal layers [4], [10], [11], differentiation of hESC in monolayer culture with high levels of activin A and bone morphogenetic protein 4 (BMP4) which yielded >30% cardiomyocytes [12], and the formation of human embryoid bodies (hEB) with growth factor supplementation resulting in 23–60% of hEB contracting [13], [14], [15] or suspension in END2 conditioned medium resulting in ∼12–70% hEB contracting [10], [14]. These techniques are all limited in their capacities for scale-up due to inherent low-throughput design, poor differentiation yields, and the use of expensive reagents. Most importantly, there is great inconsistency in differentiation efficiency between various hESC lines. This variability is likely a function of genetic and epigenetic differences between hESC lines [16], [17], [18] that directly impact their cardiac differentiation capacity [19], [20], [21]. hiPSC lines exhibit even broader epigenetic diversity [22] which may additionally limit their cardiac differentiation capacity [8]. Therefore, existing cardiac differentiation protocols developed using select hESC lines with propensities toward cardiac differentiation may not be applicable to genetically and epigenetically diverse patient-specific hiPSC lines. These limitations highlight the need for a reproducible, fully optimized and universally applicable differentiation system capable of overcoming the interline variability that commonly exists amongst human pluripotent stem cells (hPSC). As yet, no cardiac differentiation system optimized specifically for hiPSC has been demonstrated. In addition to poor differentiation yields, another limitation of hiPSC for cardiac drug testing, disease modeling or cellular therapies involves the caveats associated with generating hiPSC using retroviruses or lentiviruses. Despite overall silencing of integrated retroviral and lentivector promoters during hiPSC generation, low level expression of viral transgenes or vector promoters has the potential for insertional mutagenesis or malignant transformation following cardiac differentiation [23].

We hypothesized that undifferentiated hPSC growth rate, hEB formation, media formulation, and exposure to growth factors during cardiac differentiation could all be systematically optimized to improve cardiac differentiation. We identified and rigorously optimized >45 variables that affect the experimental variation of cardiac differentiation in genetically diverse hPSC lines. We used this data to develop a universal cardiac differentiation system that has an average efficiency of 94.7±2.4% hEB contracting for a diverse repertoire of hESC lines and hiPSC lines, including those generated from fetal fibroblasts using lentiviral methods, or neonatal CD34^+^ cord blood cells and mature-donor dermal fibroblasts using non-viral episomal-based methods.

## Results

### A systematic strategy for sequentially optimizing cardiac differentiation

**Figure 1:**
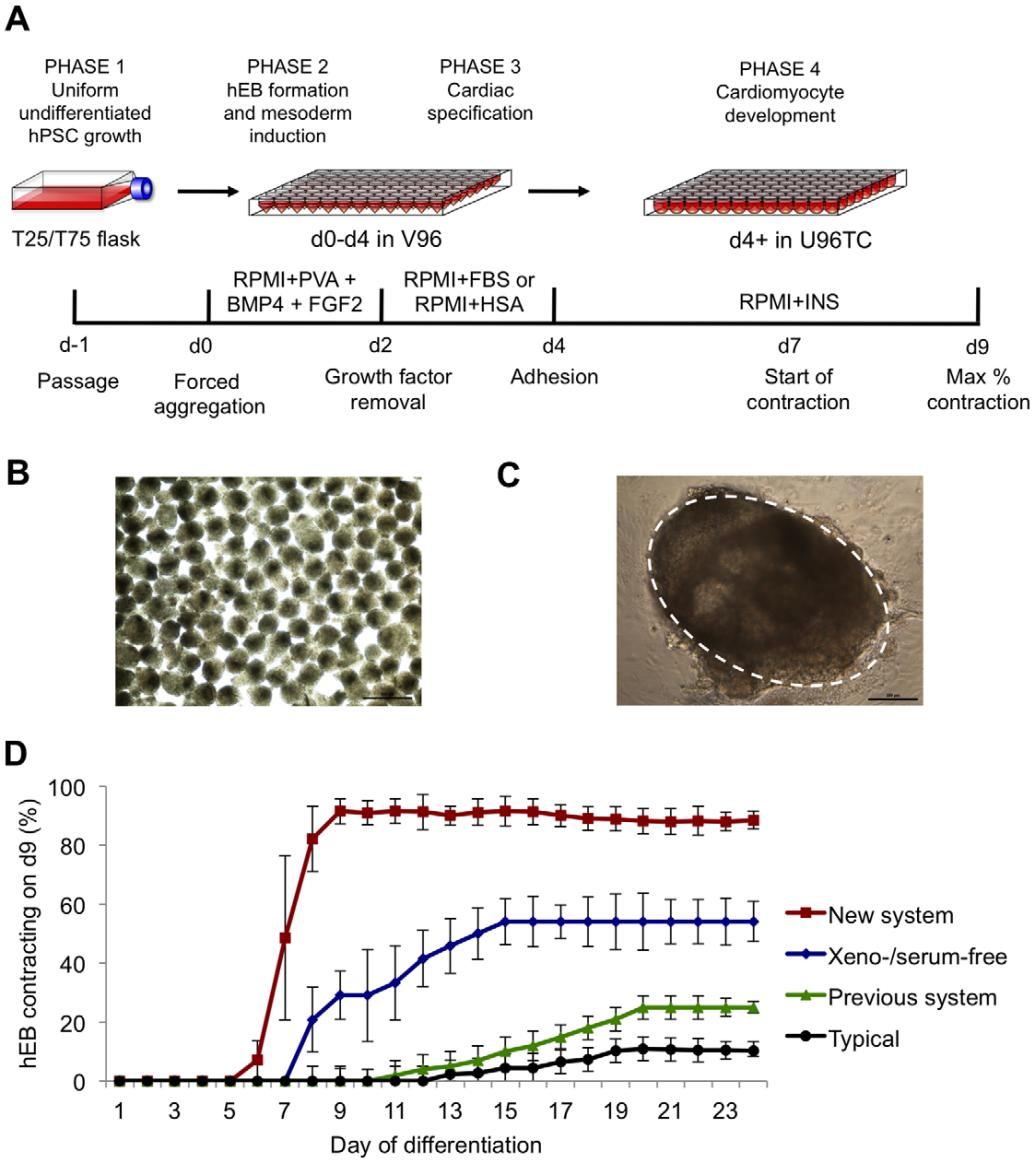
Systematic optimization of cardiac differentiation of human pluripotent stem cells. (**A**) Schematic of the optimized cardiac differentiation system demonstrating: Phase 1, uniform growth of hESC/hiPSC as monolayers. Phase 2 (d0–d2), aggregation of 5000 single cell hESC or hiPSC in chemically defined RPMI+PVA medium in V96 plates. Phase 3 (d2–d4), cardiac specification using FBS or hSA containing medium. Phase 4 (d4+), cardiac development, hEB are allowed to adhere to U96 tissue culture treated plates in RPMI+INS. (**B**) Typical d2 hEB formed using the forced aggregation procedure in RPMI-PVA to demonstrate homogeneity in hEB size. Scale bar = 500 µm. (**C**) Typical d9 contracting hEB formed using the optimized cardiac differentiation method with the contracting area circled, note minimal fibroblast outgrowth. Scale bar = 200 µm. (**D**) Efficiency of generation of contracting hEB produced in this system (New system, *n* = 48), with comparisons to xeno- and serum-free conditions (Xeno-/serum-free, *n* = 5), our previous method (Previous system, *n* = 7), and typically used methods (Typical, *n* = 9). Error bars, ± S.E.M.

To improve the efficiency and reproducibility, and to reduce the known interline variability of cardiac differentiation [15], [20], [21], we analyzed existing cardiac differentiation strategies [6],[12],[13],[14],[15],[24], identified >45 possible experimental variables (Figure S1), and initiated a strategy for systematically optimizing the cardiac differentiation of hPSC. We employed our previously described forced aggregation cardiac differentiation system, which efficiently forms uniform homogeneous hEB from known numbers of cells [15] (Figure S2A). For initial system development, we used the hESC line H9 (WA09). We divided our cardiac differentiation system into four distinct phases for rigorous, systemic optimization (Figure 1A): phase 1, uniform undifferentiated hPSC growth; phase 2, hEB formation/mesoderm induction; phase 3, hEB cardiac specification; phase 4, contracting cardiomyocyte development. We used a contracting hEB assay to sequentially assess improvements in cardiac differentiation in each of these four phases by counting the number of hEB contracting after nine days of differentiation (Figure S2B). The final fully-developed system (Figure 1A and Figure S2C) reproducibly formed homogeneous H9 hEB (Figure 1B) which began contracting at a significantly (p<3×10^−10^) improved efficiency of 91.2±1.9% contracting hEB in an accelerated time period of only 9 days of differentiation, compared to an average efficiency of 10.4±6.8% contracting hEB in 20 days using traditional methods (Figure 1D). Additionally, in contrast to prior methodologies that produce only rare, focused areas of contracting cells at the periphery of the hEB, our optimized differentiation method produced robust and forceful contractions within the entire hEB (Figure 1C and Movie S1).

### Efficient generation of vector and transgene-free hiPSC from CD34^+^ cord blood and adult fibroblasts

**Figure 2:**
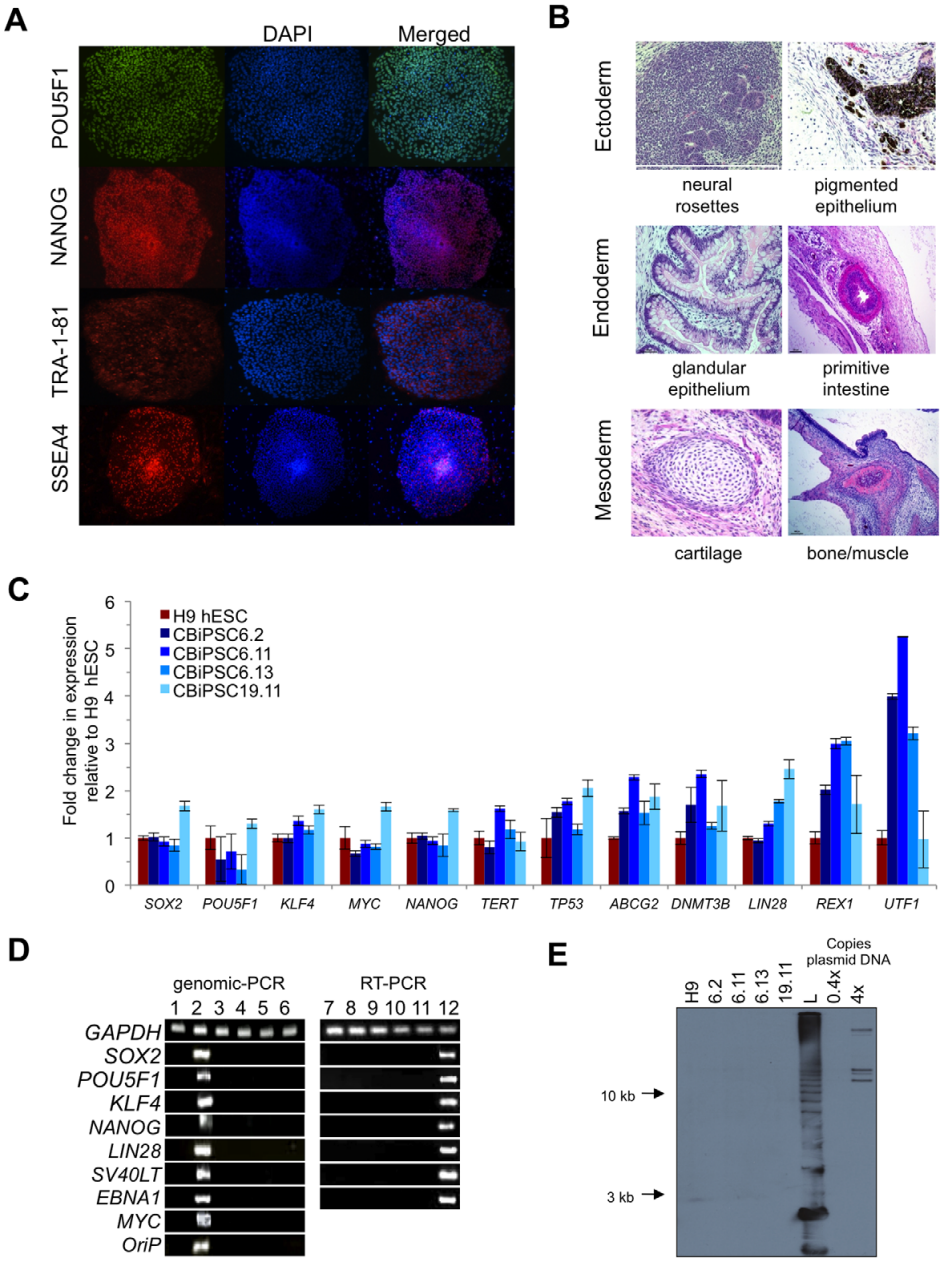
Generation of non-viral hiPSC from CD34^+^ cord blood progenitors. (**A**) Representative immunocytochemistry of pluripotency markers POU5F1 (OCT4), NANOG, TRA-1-81, and SSEA4 in hiPSC line CBiPSC6.2 after >20 passages. (**B**) Representative hematoxylin and eosin staining of teratoma sections derived from CBiPSC6.2 after >20 passages demonstrating ectodermal, endodermal and mesodermal lineage differentiation. All CBiPSC clones in these studies formed similar teratomas. (**C**) Real-time RT-PCR studies p15 CBiPSC lines for endogenous pluripotency genes using primers that distinguish endogenous expression from transgenes (see Methods). (**D**) The presence of plasmid transgene sequences examined by PCR at p11 in CBiPSC6.2, CBiPSC6.11, CBiPSC6.13, CBiPSC19.11 (lanes 3–6, respectively) and negative control H9 hESC (p48) (lane 1) compared to positive control early cultures from p2 (lane 2). RT-PCR analysis of selected plasmid sequences in p11 CBiPSC6.2, CBiPSC6.11, CBiPSC6.13, CBiPSC19.11 (lanes 8–11, respectively) and negative control H9 hESC (lane 7) and p2 cultures (lane 12). (**E**) Genomic Southern blot analysis for episomal vector backbone integration in lines CBiPSC6.2, CBiPSC6.11, CBiPSC6.13, CBiPSC19.11 (p15) (lanes 2–5, respectively), H9 hESC (p55) (lane 1). Combination 6 episomal vector DNA was diluted as positive control to the equivalents of 0.4 and 4 integrations per haploid genome (0.4× and 4×). L: 1 kb plus ladder. These studies were also conducted for non-viral adult fibroblast-derived hiPSC lines iPSCWT2 and iPSCWT4 with similar results (Machairaki *et al.*, in preparation).

To avoid potential clinical caveats of cardiomyocytes differentiated from retroviral or lentiviral derived-hiPSC (*e.g.*, insertional mutagenesis), we generated several non-viral, transgene-free hiPSC for cardiomyocyte differentiation using a three plasmid, seven-factor (SOKMNLT; *SOX2, OCT4 (POU5F1), KLF4, MYC, NANOG, LIN28, and SV40L T antigen*) EBNA-based episomal system [25]. These hiPSC cell lines were generated from adult fibroblasts (iPSCWT2, iPSCWT4), and CD34^+^ cord blood cells (CBiPSC6.2, CBiPSC6.11, CBiPSC6.13, and CBiPSC19.11). hiPSC lines expressed high levels of pluripotency markers by immunocytochemistry (Figure 2A) and real-time qRT-PCR (Figure 2C), and formed trilineage teratomas when injected into murine recipients (Figure 2B). Lack of genomic episomal vector or transgene integration and expression was demonstrated by Southern blot, genomic PCR, and RT-PCR studies of plasmid backbone and transgene sequences (Figure 2D–E). All non-viral hiPSC evaluated in these studies expressed similar levels of pluripotency markers, formed similar teratomas, and were demonstrated to be transgene- and vector-free. Full details of the derivation and characterization of these non-viral hiPSC are described in Methods. In comparison to episomal reprogramming of adult fibroblasts [25], CBiPSC from plasmid-nucleofected CD34^+^ cord blood cells emerged rapidly in 14–21 days at extremely high frequencies (average 1.4% efficiency; e.g. 300–1450 alkaline phosphatase^+^ TRA-1-81^+^ colonies per million CD34^+^ input cells; range 0.4%–3.6% efficiency; *n* = 5). These efficiencies were comparable to previously reported ‘high efficiency’ non-integrating reprogramming systems using either the same EBNA1-based episomal plasmid system we used (0.1–1% efficiency, [26]), or alternatively, synthetic mRNA-based reprogramming protocols (0.6–4.4% efficiency, [27]). Below we describe our approach for the systematic optimization of each of the four phases of our highly efficient cardiac differentiation system in various hPSC lines.

### Phase 1: Defined single-cell culture promotes uniform growth of hPSC lines

**Figure 3:**
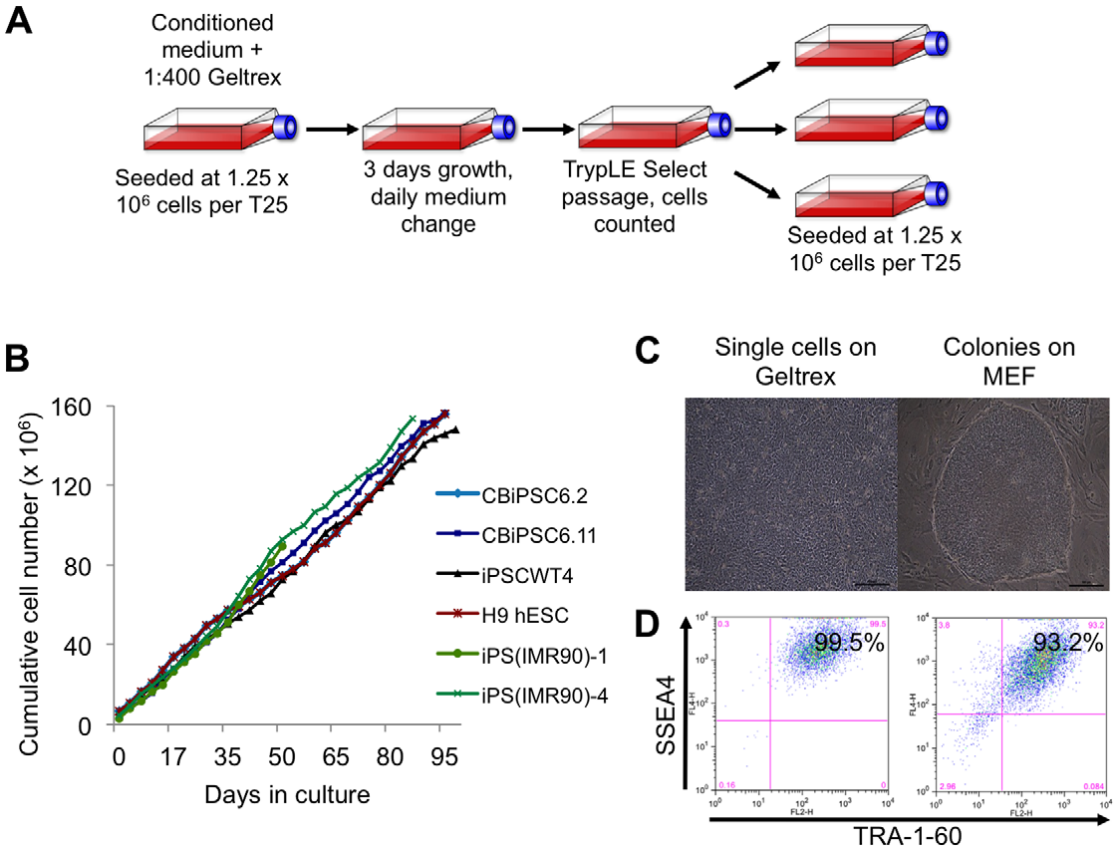
Controlled growth of hPSC lines for reproducible cardiac differentiation. (**A**) Schematic of monolayer hESC/hiPSC culture technique. This monolayer technique uses conditioned medium prepared in a defined manner, single-cell passaging, automated cell counting, plating cells at a known density, and passaging every three days. (**B**) Stable growth rates of H9 hESC, viral fetal fibroblast-derived hiPSC lines iPS(IMR90)-1 and iPS(IMR90)-4, non-viral CD34^+^ cord blood-derived hiPSC lines CBiPSC6.2, CBiPSC6.11, and non-viral adult fibroblast-derived hiPSC line iPSCWT4. (**C**) The homogenous culture phenotype seen when culturing H9 hESC as feeder-free monolayers (left), compared in comparison to typical colony morphology of cells grown in co-culture with MEF (right). (**D**) Comparable SSEA4 and TRA-1-60 expression of H9 hESC growing as monolayer cultures (left) or colonies grown in co-culture with MEF (right).

We first hypothesized that promoting uniform growth of undifferentiated hPSC lines is critical for subsequent reproducible differentiation and for the derivation of a universal cardiac differentiation system. To conform all hPSC lines to one universal culture method, we adapted all lines to feeder-free monolayer growth [28] on the basement membrane matrix Geltrex in conditioned medium [29]. In this system, cells were enzymatically-passaged to single cells on a rigid timescale of every three days, counted using an automated cell counter, and plated at fixed cell densities (Figure 3A). This approach resulted in controlled, reproducible growth of four hESC lines (H1, H9, ES03, and SI-233), as well as seven hiPSC lines including lentiviral fetal lung fibroblast-derived hiPSC lines iPS(IMR90)-1 and iPS(IMR90)-4, as well as our own episomal CD34^+^ cord blood-derived hiPSC lines (CBiPSC6.2, CBiPSC6.11 and CBiPSC6.13) and non-viral adult dermal fibroblast-derived lines (iPSCWT2 and iPSCWT4) for over 30 passages (Figure 3B). Cells cultured in this manner maintained high expression levels (>99%) of pluripotency markers SSEA4 and TRA-1-60 (Figure 3C).

### Phase 2 (d0–d2): A chemically defined medium supplemented with BMP4 and FGF2 accelerates mesoderm induction

To accelerate mesoderm induction in the second phase of this system, we optimized hEB formation via forced aggregation [15] by systematically testing 21 published hESC and mESC differentiation and pluripotent culture media formulations. We found that the chemically defined media formulation originally devised by Wiles and Johansson [30] formed the most reproducibly homogeneous hEB via forced aggregation (Figure S3A). By subtraction analysis we found that only three of the components of this media (basal media, PVA, and insulin) were necessary for forced aggregation hEB formation (Figure S3B). We next tested the individual supplements to this media formulation in an effort to further enhance cardiac differentiation. The final d0–d2 media formulation that gave the most efficient cardiac differentiation is described as RPMI+PVA (Table 1). The combination of the growth factors BMP4 and FGF2 was determined to be necessary for optimally efficient cardiac differentiation (Figure S4A, B). Other growth factors including NODAL, activin A, TDGF1, BMP2, BMP6, TGFB, IGF1, IGF2 and WNT3A were each individually titrated between 1–100 ng mL^−1^, and none were found to have the same efficacy as BMP4 (data not shown). We also assessed the effects of cell handling prior to and during forced aggregation. Cardiac differentiation of hEB made from monolayers of pluripotent cells passaged one day prior to aggregation was more efficient (93.8±3.3% contracting hEB) than the differentiation of those passaged 2 days (46.9±4.1%), or 3 days (26.0±8.0%) earlier (Figure S5H). Centrifugal force was not required for hEB formation using this media formulation (Figure S5F). Other aggregation hEB formation techniques such as AggreWell (StemCell Technologies) [31] or ‘Shrinky-dink’ methods did not result in cardiac differentiation [32] (data not shown).

**Table 1:** Final optimized media formulations for each of the three steps of cardiac differentiation.

Phase 2 (d0–d2) medium RPMI-PVA	Phase 3 (d2–d4) medium RPMI-FBS	Phase 4 (d4+) medium RPMI-INS
RPMI 1640	RPMI 1640	RPMI 1640
400 µM 1-thioglycerol	400 µM 1-thioglycerol	400 µM 1-thioglycerol
4 mg mL^−1^ PVA	20% FBS or human serum	10 µg mL^−1^ hr-Insulin
10 µg mL^−1^ hr-Insulin	**Xeno-/serum-free variant**	1× chemically defined lipids
25 ng mL^−1^ BMP4	RPMI 1640	
5 ng mL^−1^ FGF2	400 µM 1-thioglycerol	
1× chemically defined lipids	1× chemically defined lipids	
1 µM Y-27632	5 mg mL^−1^ HSA	
5% O_2_	280 µM L-ascorbic acid	

The basal medium RPMI 1640 was found to be most successful for cardiac induction during phase 2 and phase 3 and therefore was maintained for phase 4. The addition of the thiol 1-thioglycerol was found to be essential for hEB formation and survival. High concentrations of PVA enhanced hiPSC hEB formation. Insulin was essential for hEB formation. The combination of BMP4 and FGF2 was optimal for mesoderm specification whilst FBS or human serum was essential in phase 3 for <90% contracting hEB differentiation efficiency. The addition of lipids in phase 2 and 4 enhanced hEB survival. A low concentration of Y27632 (ROCK inhibitor) enhanced the reproducibility of hEB formation. The xeno-/serum-free variant of the phase 3 medium required the addition of HSA and L-ascorbic acid to maintain cardiac differentiation and produces ∼60% of hEB contracting by d15 of differentiation.

### Phase 3 (d2–d4): Efficient cardiac specification is inhibited by insulin and potentiated by FBS or human serum

Once H9 hEB formation and mesodermal induction was reproducibly maximized, we focused on optimizing the third phase of differentiation: cardiac specification. We found that supplementation with 20% FBS was essential for efficient cardiac differentiation (Figure S6A) and that the supplier of FBS did not impact cardiac differentiation (Figure S6B). Additionally we found that replacement of FBS with human serum maintained this same high efficiency differentiation. Finally, we discovered that the supplementation of this d2–d4 step with any level of insulin (which was essential for the preceding phase 2 d0–d2 hEB formation step) completely abrogated cardiac specification (Figure S6H).

### Phase 4 (d4+): hEB adherence and chemically defined media enhances final cardiomyocyte differentiation

Once we completed formulation of the optimal cardiac specification media, we focused on enhancing the final steps of cardiomyocyte differentiation and maintenance. We found that adherence onto tissue culture treated plates on d4 enhanced subsequent cardiomyocyte development, whilst adherence before this time-point almost completely ablated contraction (Figure S5D). Unlike the third phase media formulation, the media formulation for this fourth phase was not dependent on factors contained in FBS (Figure S7A). Moreover, once contraction had begun, we found that hEB could be successfully maintained in a variety of media (e.g. RPMI+FBS or RPMI+PVA or simple RPMI+INS (Table 1)) for at least 3 months with continuous contraction. In both RPMI+PVA and RPMI+INS media the hEB formed substantially less fibroblast outgrowth than seen when using RPMI-FBS. A summary of the optimal media and factors for each phase is provided in Table S2.

### Polyvinyl alcohol (PVA) and physiological oxygen tension synergize to induce highly efficient cardiac differentiation of hiPSC

**Figure 4:**
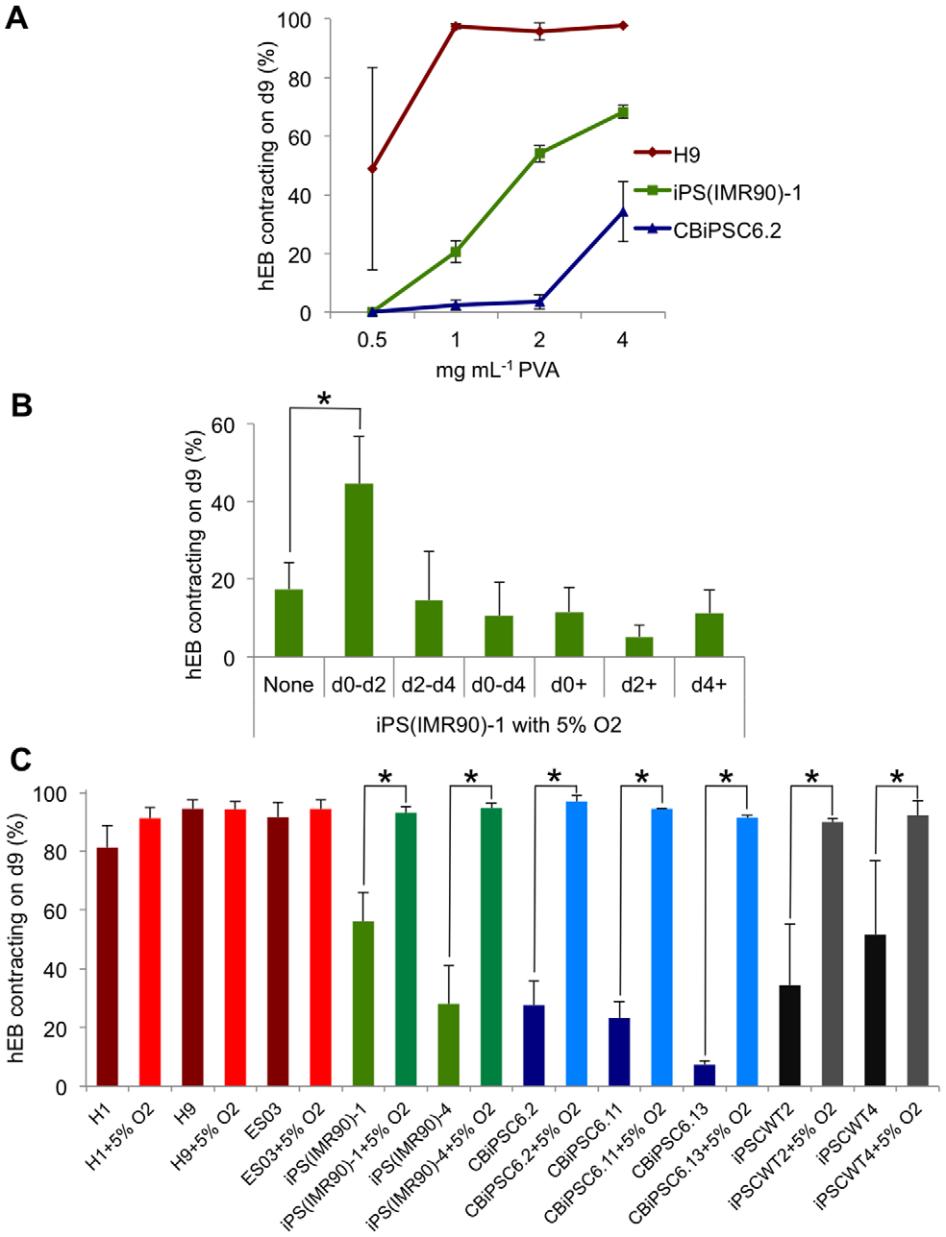
PVA supplementation and staged exposure to physiological oxygen eliminates interline variability of cardiac differentiation. (**A**) Increasing the concentration of PVA from 1 mg mL^−1^ to 4 mg mL^−1^ PVA in the d0–d2 media formulation enhanced the differentiation of viral fibroblast-derived hiPSC line iPS(IMR90)-1, and non-viral cord blood-derived hiPSC line CB-iPSC6.2, whilst not affecting H9 hESC (*n* = 3). (**B**) Exposure of iPS(IMR90)-1 hEB to physiological (5%) oxygen tensions from differentiation d0–d2 also enhanced cardiac differentiation (*n* = 3, p<0.005). Identical conditions did not improve already highly efficient H9 hESC differentiation. (**C**) Combining both physiological oxygen tension and 4 mg mL^−1^ PVA between d0–d2 eliminated interline differentiation variability in hiPSC derived using both viral- and non-viral-techniques (*n*≥3, p<0.005). Error bars, ± S.E.M.

We next assessed the performance of our H9 hESC-optimized cardiac differentiation system, using the hESC lines H1, ES03 and SI-233, and we found that similarly high efficiencies of cardiac differentiation could be achieved. However, this system produced only low levels (2.5–20.5%) of contracting hEB for various hiPSC lines (Figure 4A, C). Attempts to re-optimize the dose-response dependant variables from phases two and three using the hiPSC line iPS(IMR90)-1 resulted in identical optimal conditions to those we had found for H9, albeit at lower efficiencies. We noted that the main impediment to efficient hiPSC differentiation was that hiPSC-derived hEB were substantially less stable and robust than those formed from hESC lines. To improve this hEB instability we tested the inclusion of extracellular matrix proteins (1∶100 Matrigel or laminin-511 and nidogen-1) [33] during hEB formation (at d0–d2, d2–d4 or d0–d4), but we found that these proteins completely abrogated cardiac differentiation (data not shown). However, the inclusion of increasing concentrations of the synthetic polymer polyvinyl alcohol (PVA; from 1 mg mL^−1^ to 4 mg mL^−1^) was highly effective in increasing the percentage contraction of iPS(IMR90)-1 hiPSC hEB from 20.6±3.7% to 68.3±2.3%, and CBiPSC6.2 hEB from 2.5±1.8% to 34.2±10.2% (Figure 4B). Concentrations of PVA above 4 mg mL^−1^ were less effective at improving cardiac differentiation (Figure S4K). This increased PVA concentration did not affect hESC experiments that already consistently differentiated at high (>91.4%) efficiencies at 1, 2, or 4 mg mL^−1^ (Figure 3A). We also assessed the effects of physiological oxygen tensions on differentiation efficiency by subjecting differentiation cultures to 5% O_2_ at timed intervals (i.e. during d0–d2, d2–d4, d4 onwards, or combinations thereof). These experiments revealed that 5% O_2_ between d0–d2 significantly (p<0.028) enhanced the differentiation of all hiPSC lines tested but had little effect on the already high efficiency of hESC differentiation (Figure 4B, C). The combined use of higher concentrations of PVA and timed exposure to physiological oxygen tensions significantly (p<0.04) enhanced cardiac differentiation and allowed each of the seven hiPSC lines we tested to achieve cardiac differentiation with an average efficiency 94.7±2.4% of hEB contracting (Figure 4C and Movie S2).

### hPSC-derived cardiomyocytes display functional cardiac properties including reproducible electrophysiological profiles and drug responsiveness

**Figure 5:**
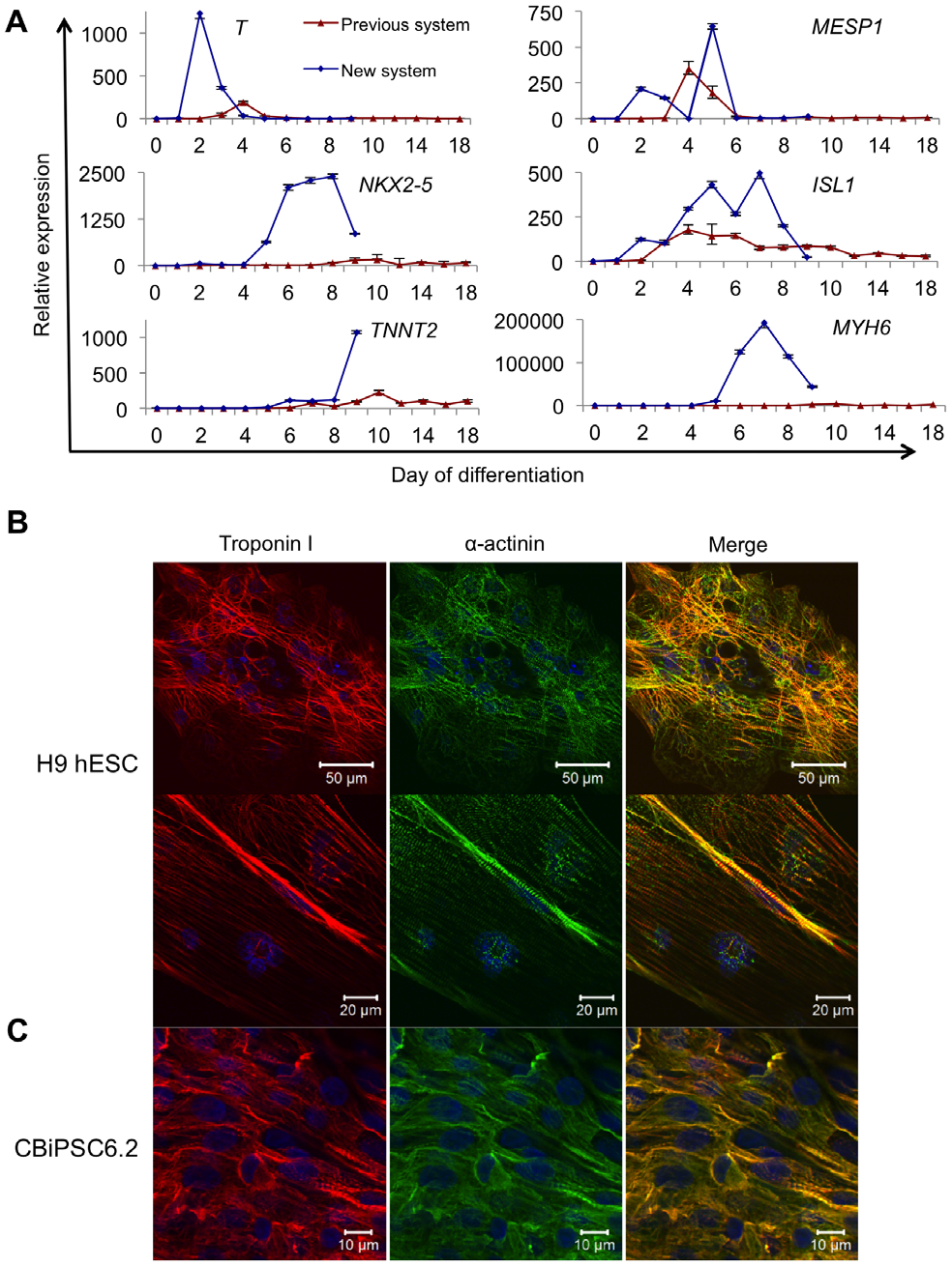
Characterization of hPSC cardiomyocyte differentiation. (**A**) Comparison of real-time RT-PCR for markers of mesoderm (*T, MESP1*), cardiac progenitors (*NKX2.5, ISL1*), and cardiomyocytes (*TNNT2, MYH6*) during hESC differentiation using either the Previous system or New system. Analysis was performed using the ^ΔΔ^Ct method with relative expression calculated using d0 of differentiation (hESC samples) as baseline. 18S RNA expression was used for normalization. Primers are shown in Table S1. (**B**) Immunocytochemistry for cardiac markers in hEB differentiated from H9 hESC. Troponin I (red), α-actinin (green) and DAPI (blue) at low power (top panels) demonstrating unaligned striations throughout the hEB and higher power (lower panels) demonstrating area of aligned striation. (**C**) Immunocytochemistry for cardiac markers in hEB differentiated from CBiPSC6.2.

**Figure 6:**
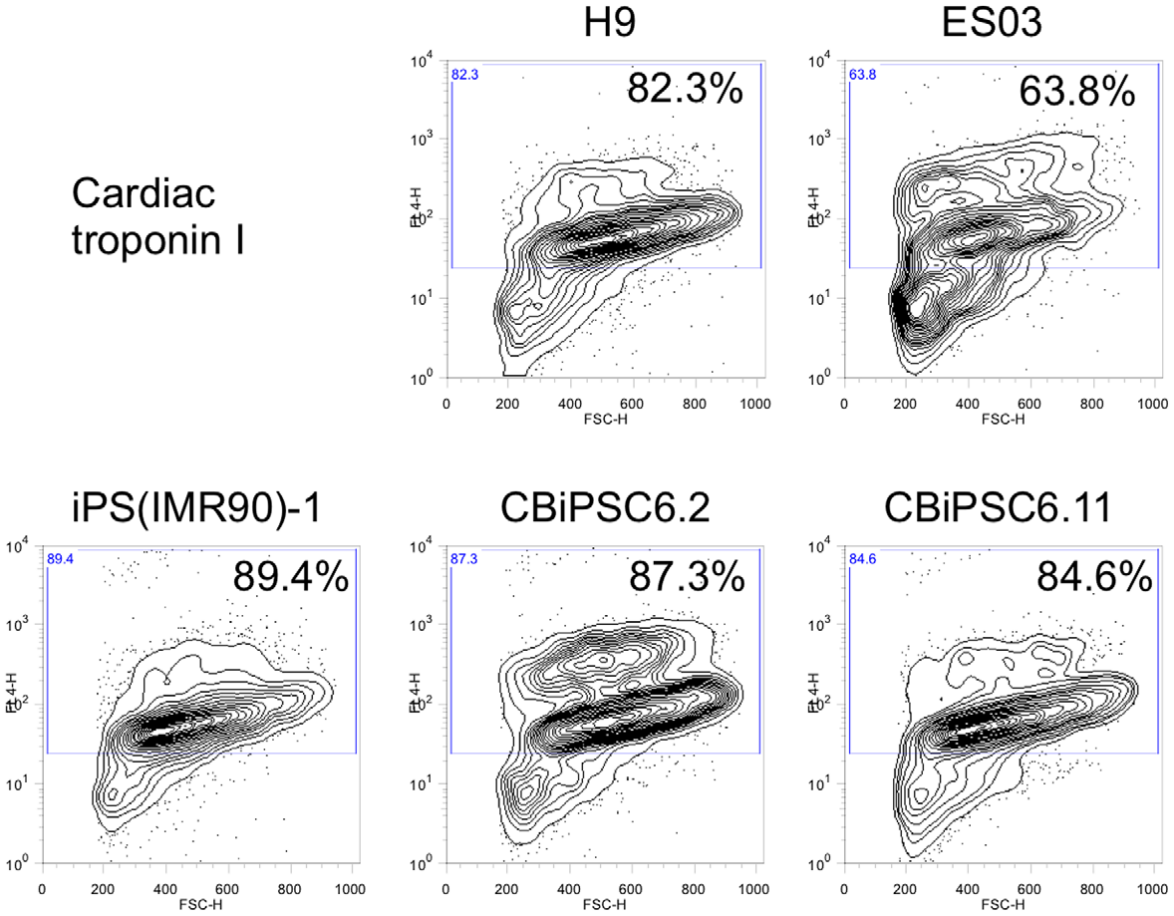
Intracytoplasmic flow cytometry analysis of cardiomyocyte marker expression. Expression of cardiac troponin I in contracting hEB from differentiated H9 hESC, ES03 hESC, viral fibroblast-derived line iPS(IMR90)-1 hiPSC, and non-viral CD34+ cord blood-derived hiPSC lines CBiPSC6.2 and CBiPSC6.11. For each sample the entire U-well contents of one 96-well plate was enzymatically digested into a single cell suspension and analyzed by flow cytometry.

Using real-time RT-PCR analysis, we established that hEB differentiated using this optimized system progress through the normal developmental stages of cardiac lineage gene expression (Figure 5A). Our data demonstrated that, compared to our previous system [15], the relative peak in mesodermal gene expression (assayed by expression of *T (Brachyury)* and *MESP1*) was substantially increased (2–6-fold) and accelerated from 4 to 2 days. Expression of cardiac progenitor markers (*NKX2-5* and *ISL1*) and terminal cardiac markers (*TNNT2* and *MYH6*) was substantially enhanced (5–2500-fold) (Figure 5A). We also analyzed the expression of the cardiac structural proteins α-actinin (ACTN2) and cardiac troponin I (TNNI3) using immunocytochemistry, and we showed that cardiomyocytes differentiated from H9 hESC and CBiPSC6.2 formed striated sarcomeres (Figure 5B, C). Gap junction formation was also demonstrated by expression of CX43 (GJA1; data not shown). Finally, intracytoplasmic staining and flow cytometry analysis of various hESC and hiPSC lines demonstrated that the entire contents of each 96-well consisted of 64–89% cardiac troponin I (TNNI3)^+^ cardiomyocytes (Figure 6).

**Figure 7:**
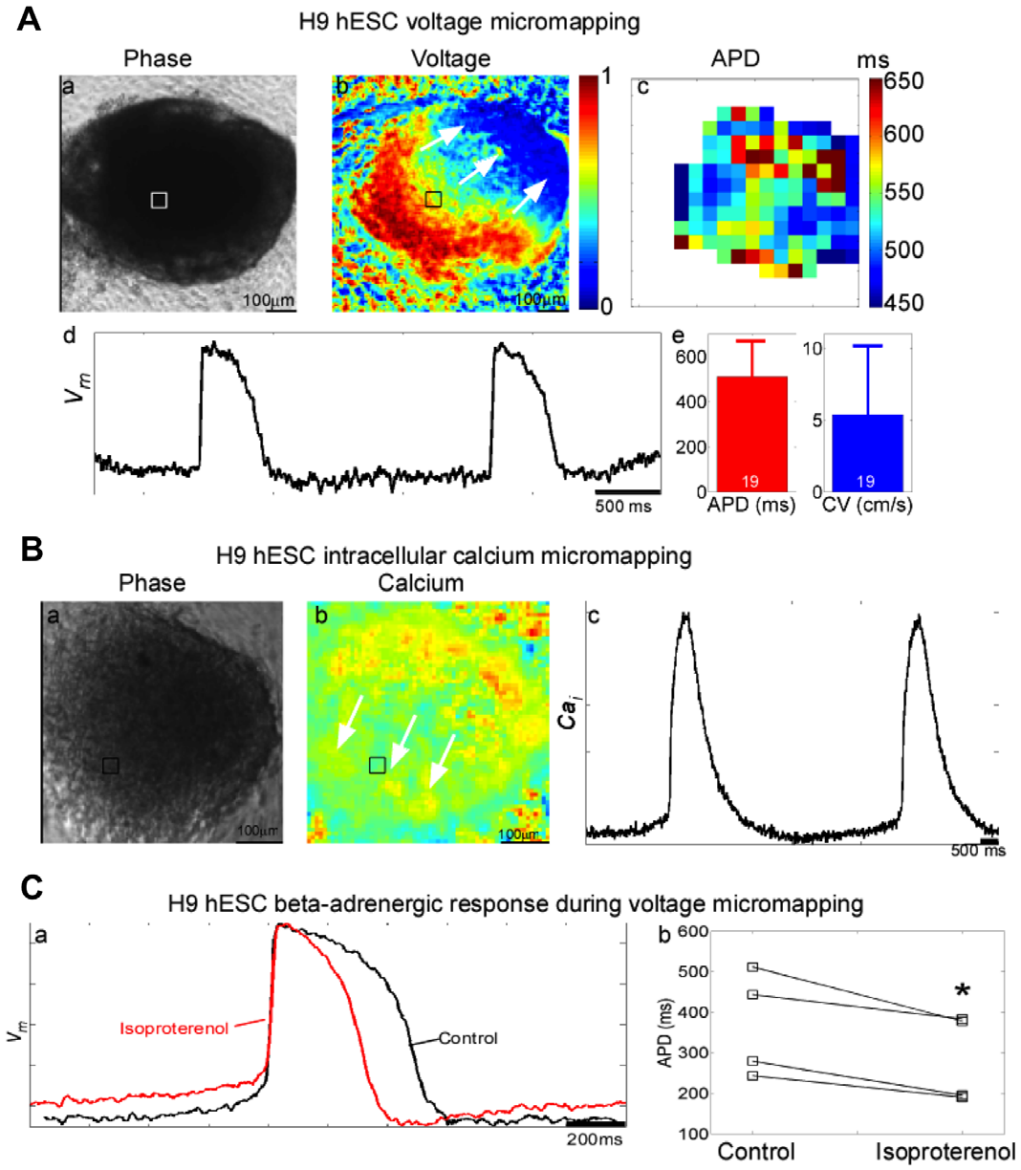
Electrophysiological characterization H9 hESC contracting hEB using optical mapping. (**A**) Voltage micromapping. a, Phase contrast image of H9 hEB at 4× magnification. b, Voltage activation map (arrows indicate direction of electrical wave propagating across hEB). c, Action potential duration (APD) map. d, Representative transmembrane potential (V_m_) trace at position denoted by the small square in a and b during 0.5 Hz field stimulation. e, Mean APD and conduction velocity (CV) measurements from 19 hEB (error bars represent ± s.d.). Coefficient of variation (COV, population s.d. divided by mean) for APD was 0.30 and for CV was 0.88 across hEB population. COV within an individual hEB was calculated from multiple APD measurements across all of the recording sites for that hEB (panel A, c) and was 0.042±0.030 (s.d.) when averaged across 19 hEB. (**B**) Intracellular calcium micromapping. a, Phase map of hEB at 6× magnification. b, Calcium map (arrows indicate direction of propagating calcium wave). c, Representative intracellular calcium (Ca_i_) trace at position denoted by the box in a and b. (**C**) The beta-adrenergic agonist isoproterenol shortened the mean APD in all 4 hEB by an average of 23±8 ms (mean ± s.d.). * indicates p≈0.01 in a paired Student's t-test.

**Figure 8:**
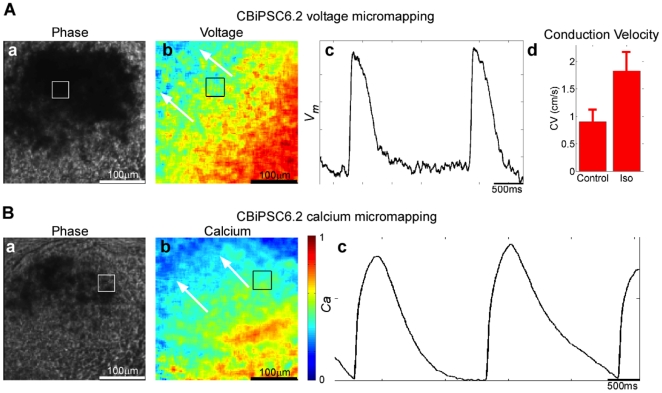
Electrophysiological characterization non-viral cord blood-derived hiPSC contracting hEB differentiated using optical mapping. (**A**) Voltage micromapping. Phase image of CBiPSC6.2 hEB at 4× magnification. b. Voltage map, hEB were stained with di-4-ANEPPS (a voltage-sensitive fluorescent dye), and electrically field-stimulated at the same pacing rate (arrows indicate direction of electrical wave propagating across hEB). c. Action potentials during 0.5 Hz field stimulation. d. Average conduction velocity (CV) measurements (mean ± standard deviation) for control and 15 min after addition of 50 µM isoproterenol during 0.5 Hz field stimulation. (**B**) Calcium micromapping. a. Phase image of beating hEB. b. Calcium map (arrows indicate direction of calcium wave propagating across beating hEB). c. Representative calcium transient (Ca) waveforms during 0.5 Hz field stimulation. Box in a and b denote site of recording in c.

Using optical mapping methods, we next evaluated the electrophysiological properties of cardiomyocytes generated from H9 hESC with this system. The hEB were either mechanically dissected for micromapping or dissociated into single cells and plated as a confluent monolayer for macromapping [34]. hEB and monolayers were then stained with either voltage- or calcium-sensitive dye and optically mapped to visualize spontaneous activity and response to electrical field stimulation (Figure 7A, 8A, S8). Replicates of voltage micromapping experiments (*n* = 19) demonstrated reproducible action potential duration and conduction velocities (Figure 7B and Movie S3). Optical mapping of intracellular calcium demonstrated a physiological calcium transient (Figure 7B, 8B, S8 and Movie S4). To assess cardioactive drug responsiveness, 20 µM isoproterenol or 100 µM pinacidil was added to cardiomyocyte monolayers or hEB to test for beta-adrenergic stimulation response and the presence of functional K_ATP_ channels, respectively. Both drugs produced a shortening of the action potential (Figure 7C and Figure S8A). 20 µM isoproterenol was demonstrated to induce increase in conduction velocity in CBiPSC6.2 hEB (Figure 8B). Functional electrical coupling within a cardiomyocyte monolayer (Figure S8A) and between a pair of hEB (Figure S8B) was demonstrated by voltage mapping. Contracting hEB derived from the hiPSC lines CBiPSC6.2 (Figure 8) and iPSCWT2 (data not shown) were also tested in the same manner and yielded similar results.

### Cardiac differentiation can be performed using xeno-free and serum-free conditions

To further maximize the ultimate clinical utility of our method, we also conducted parallel optimization experiments that focused on the complete elimination of serum during the third phase of differentiation (d2–d4). We formulated a serum-free optimal media containing human serum albumin (HSA), L-ascorbic acid, and lipids (Table 1 and Figure S9) and found that this formula, used from day 2–4 to replace the FBS or human serum containing media produced 64.8±3.3% of hEB contracting by d15 of differentiation (Figure 1D). Additional supplementation with DKK1 and VEGFA_165_ [13] did not further enhance differentiation (data not shown).

## Discussion

We have demonstrated that by systematically and rigorously optimizing culture conditions, we could significantly improve cardiac differentiation efficiency to an average of 94.7±2.4% of hEB contracting from four hESC and seven hiPSC lines. The variation in cardiac differentiation potential among different hPSC lines cultured under similar conditions has been well documented [15], [20], [21], [35]. Although robust cardiac differentiation in select hPSC lines has been reported [9], [12], [13], [24], highly efficient cardiac differentiation of multiple independently derived hESC and hiPSC lines using a single technique has thus far not been possible. One reason for this difficulty may be that significant variability exists in the innate response to cardiac inductive factors among different hESC lines [8], [15], [20], [35]. Previous cardiac differentiation systems may simply leverage the innate cardiac potential of specific hESC lines, and therefore not be suitable for other hESC lines with differing propensities. Such cardiac differentiation systems may be even less effective for hiPSC differentiation, as these cell types have been demonstrated to possess wider variation in gene expression [36].

In developing a universal cardiac differentiation system, we evaluated and tested the strengths of multiple published protocols with a multivariate strategy. Although we initially favored a monolayer differentiation based technique [12] due to its simplicity and reproducibility, monolayer based differentiation systems have been demonstrated to be less responsive to cardiac inductive factors than in hEB systems [37]. Our previous data demonstrated that hEB could be formed from feeder-free single-cell hESC using forced aggregation in chemically defined media (CDM) [30]. The use of CDM enhanced the effectiveness of recombinant growth factors due to the exclusion of FBS or BSA [38]. We tested a large number of mesodermal morphogens from the NODAL, BMP4, and WNT signaling cascades for cardiogenic potential. However, only the combination of BMP4 and FGF2 was suitable for highly efficient cardiac differentiation. Indeed, BMP4 is a known potent mesoderm morphogen in hESC [39], with a brief temporal window of effectiveness for mesendoderm induction (d1–d2) [40]. Furthermore, BMP4 and FGF2 synergize to promote mesoderm induction [39], [41]. We noted that in the d2–d4 stage, insulin completely ablated cardiac differentiation even at 5 µg mL^−1^. The negative effect of insulin on cardiac differentiation has been previously reported with an effective window from d3 onwards [14]. Freud *et al.* demonstrated that insulin, acting primarily via IGF1R and PI3K/Akt, has an inhibitory effect on each of d0–d5 of cardiac differentiation, due to a redirection of cardiogenic mesoderm to neuroectoderm [10]. We have defined this negative effect to a small temporal window of d2–d4. One reason for the lack of negative effect of insulin in our system between d0–d2 is that it was required during this period for successful hEB formation (Figure S3B). We also discovered that either 20% FBS or human serum was essential for phase 3 (d2–d4) of this system, indicating that additional serum factors are required at this stage. This requirement of FBS for cardiac differentiation has previously been demonstrated [42]. Although FBS is undefined, the 20% FBS media contains approximately 0.2–20 µg mL^−1^ of insulin [43], suggesting that cardiac differentiation efficiency may be further enhanced if the cardiogenic properties of FBS can be identified and recreated in the absence of insulin. In our defined serum- and xeno-free version of our differentiation system (Table 1 and Figure S9), replacing FBS/human serum with HSA, lipids and L-ascorbic acid [11] reduced differentiation efficiency to ∼65%, suggesting that additional factors in FBS/human serum are required at this stage. After day 4 of differentiation, we found that only a very simple media was required for the completion of cardiomyocyte specification, suggesting that once the cardiomyocyte program is initiated by sequential treatment with BMP4/FG2 followed by FBS, this final process is essentially self-specifying. Once contraction began, hEB could be maintained in a simple media formulation for extended periods, as previously described [44].

Once a system for highly efficient cardiac differentiation of H9 hESC was established, we found that our improved system overcame the well-documented variation in cardiac differentiation potential among different hESC lines [15], [20], [21], [35]. We then further applied this system to hiPSC cardiac differentiation, and noted that efficiency was initially poorer than that of hESC, as reported in other differentiation systems [8], and would require additional optimizations. These results were consistent with the notion that there are important biological differences between hiPSC and hESC. For example, it has been demonstrated that early passage iPSC retain an epigenetic memory of their somatic cell of origin which may affect lineage-specific differentiation capacity [22], [45]. Such inherent epigenetic limitations of iPSC lineage-specific differentiation have been partially overcome by the use of chromatin-modifying drugs (e.g. the demethylation inhibitor 5-azacytidine [9], [22] or the HDAC inhibitor trichostatin A [46]). In contrast, superior hiPSC cardiac differentiation efficiencies were achieved in our system without need for non-specific, toxic, and potentially mutagenic drugs. Instead, we achieved comparably high efficiencies in both hESC and hiPSC by applying our highly efficient hESC system with enhancement of the structural integrity of hiPSC hEB using PVA, and using timed exposure to physiological oxygen tensions. PVA, a common constituent of embryo culture media, is a cost-effective media additive that is used to replace the need for BSA or FBS, creating a true chemically defined media, as well as functioning as an adhesive. The adhesive properties of PVA likely enhance hiPSC hEB formation, and promoted their subsequent cardiac specification. Low oxygen tensions also have established effects for improving embryoid body formation [47], [48]. Low oxygen tensions also affect a wide range of developmental processes, including cardiogenesis, the stem cell niche, and modulation of NODAL, VEGF, WNT and NOTCH signaling [49]. In particular 5% O_2_ has been shown to induce WNT pathway signaling by enhancing β-catenin activation [49]. The positive effect of 5% O_2_ during only d0–d4 of our differentiation, and subsequent negative effect after day 4 suggests that WNT signaling may be the 5% O_2_ effector in our system. We found that the addition of the WNT signaling inhibitor DKK1 only impacted cardiac differentiation when added at d0–d2 and none of the subsequent stages (data not shown).

Electrophysiological assessment of our contracting hEB demonstrated that these hEB displayed characteristics of immature cardiomycytes. Although our electrophysiology method does not evaluate single or clusters of cells, the whole hEBs that were assessed displayed, on average, a ventricular-type electrophysiological profile. In addition, we demonstrated that each well contained a high percentage of troponin I^+^ cells. Based on this data for every 500,000 input cells, one 96-well plate produced ∼500,000 cells, and an average of 407,000 cardiomyocytes.

In summary, this system produced highly efficient cardiomyocyte differentiation from a wide variety of independently derived hESC and nonviral, non-integrated hiPSC lines. Importantly we demonstrate that the contracting cells produced using this system expressed normal cardiomyocyte markers, were capable of electrically coupling, and displayed highly reproducible electrophysiological profiles. The development of a universal cardiac differentiation protocol that can translate across multiple pluripotent stem cell lines allows immediate application to genetically diverse hiPSC lines created from patients with cardiac related diseases (e.g. long QT syndrome [50], [51]). The uniformity of the electrophysiological profiles of these cells highlights the potential for translation of this methodology to future high-throughput cardiotoxicity testing and novel drug discovery assays that can be used at various stages of drug development. The generation of cardiomyocytes from clinically safe cord blood-derived hiPSC is especially attractive since this cell source is widely available, carries relatively few somatic mutations, and could ultimately be used to create an HLA-defined stem cell bank for hiPSC generation via worldwide networks of existing cord blood banks. Overall, the development of this efficient cardiac differentiation system should greatly facilitate the utility of hiPSC-derived cardiomyocytes in drug development and cardiotoxicity screening, cardiac developmental biology and disease modeling, and contribute to the future generation of clinically safe human cardiac cells for regenerative medicine.

## Materials and Methods

### Human pluripotent stem cell culture

All tissue culture reagents were purchased from Invitrogen (Carlsbad, CA, http://www.invitrogen.com) unless otherwise stated. MEF, hESC and hiPSC cultures were maintained at 37°C, 5% CO_2_ and 85% relative humidity. Medium was changed every day on hESC and hiPSC cultures. Physiological (5%) oxygen conditions were created in a hypoxia chamber using nitrogen gas by ProOx Oxygen and ProCO_2_ CO_2_ controllers (BioSpherix, Lacona, NY, http://www.biospherix.com/). hESC lines H1 (WA01) and H9 (WA09) [52], ES03 (HES1) [53], and hiPSC lines iPS(IMR90)-1-DL-1 and iPS(IMR90)-4-DL-1 derived from fibroblasts using lentiviruses [25] were obtained from the WiCell WISC Bank (Madison, WI, http://www.wicell.org). The hESC line SI-233 [54] was obtained from Stemride International (London, UK, http://www.stemride.com). hiPSC lines CBiPSC6.2, CBiPSC6.11, and CBiPSC6.13 were derived in our lab from CD34^+^ cord blood using a modified episomal plasmid methodology described below. hiPSC lines iPSCWT2, and iPSCWT4 were derived in our lab from adult fibroblasts using a modified episomal plasmid methodology described below. All hESC lines used in these studies were approved for use by The Johns Hopkins University Institutional Stem Cell Research Oversight Committee. Pluripotent stem cell lines were initially cultured as colonies on irradiated (5000 cGy) MEF (E13.5 DR4 seeded at 2×10^4^ cells cm^−2^) in 6-well plates (Greiner Bio-One) in hESC medium consisting of DMEM-F12, 15% Knockout Serum Replacer (KSR), 1% non-essential amino acids (NEAA), 100 µM 2-mercaptoethanol and 4 ng mL^−1^ human FGF2 (R&D Systems, Minneapolis, MN, http://www.rndsystems.com) and passaged with collagenase IV. For transfer of hiPSC and hESC to monolayer culture [28] 1 confluent well of a 6-well plate was treated with TrypLE Select and single cells were passaged into a T25 flask (BD Biosciences, Bedford, MA, http://www.bdbiosciences.com) coated with a 1∶400 dilution (200 µL cm^−2^) of Geltrex in 5 mL of conditioned medium. Conditioned medium was made essentially as previously described [29]. In brief, confluent p2 MEF were irradiated (5000 cGy) and seeded at 6×10^4^ cells cm^−2^ on gelatin coated flasks in MEF medium consisting of DMEM (with Glutamine), 10% fetal bovine serum (FBS, Characterized, Hyclone, Thermo Fisher, Waltham MA, http://www.hyclone.com), 1% NEAA, 55 µM 2-mercaptoethanol. After allowing MEF to attach for 24 h cells were washed and media was replaced with 0.5 mL cm^−2^ hESC medium. Medium was conditioned for 22–26 h, pooled, filter sterilized and supplemented with an additional 4 ng mL^−1^ FGF2 and stored at −20°C. Conditioned medium was collected for 7 days. Confluent cultures were passaged every 3 days washing with PBS then treating with room temperature TrypLE Select for 1 min at 37°C. Confluent cultures were passaged every 3 days with TrypLE Select. Cells were counted with a Countess Automated Cell Counter and seeded at 1.25×10^6^ cells per T25 flask. All hPSC lines commonly grew from 1.25×10^6^ to 5×10^6^ in these 3 days.

### Generation of Vector and Transgene-Free hiPSC

EBNA-based pCEP4 vectors pEP4 EO2S EN2L (*OCT4, SOX2, NANOG, LIN28*), pEP4 EO2S ET2K (*OCT4, SOX2, SV40LT, KLF4*), pEP4 EO2S EM2K (*OCT4, SOX2, MYC, KLF4*) were obtained from Addgene (Cambridge, MA, http://www.addgene.org). *E. coli* containing the plasmids were propagated and purified using a Plasmid Maxi Kit (QIAGEN, Valencia, CA, http://www.qiagen.com). Ratios of (1∶1∶1) were mixed as the seven-factor SOKMNLT (Combination 6 [25]) and co-concentrated using a QIAquick PCR Purification Kit.

For generation of the hiPSC lines iPSCWT2 and iPSCWT4, adult fibroblasts from a normal 56 year-old female donor were obtained from the Coriell Cell Repository (Coriell, Camden, NJ, http://www.ccr.coriell.org/) and cultured in standard conditions. Two-three days following passage, cells were trypisinized and 1×10^6^ cells were nucleofected in NHDF nucleofector solution (VPD-1001, Lonza, Walkersville, MD, http://www.lonzabio.com) with 6 µg total of the three plasmids using an AMAXA II nucleofector (Lonza) and program U023. Cells were then plated on three 10 cm plates seeded with irradiated MEF (5000 cGy, E13.5 DR4 MEF seeded at 2×10^4^ cells cm^−2^) in fibroblast (MEF) medium. Nucleofection solution medium was changed after 4–6 hours. After 3 days fibroblast medium was replaced with hESC medium containing 40 ng mL^−1^ FGF2. Medium was changed every 2 days. Starting on Day 10 medium was changed every day using MEF-conditioned medium (made as above) supplemented with 40 ng mL^−1^ FGF2. After 21 days+ colonies were passaged onto fresh irradiated MEF layers, and ESC-like colonies that emerged were manually picked and expanded for further analysis, as described [55].

For generation of the non-viral CD34+ cord blood-derived hiPSC lines (CBiPSC6.2, CBiPSC6.11, CBiPSC6.13 and CBiPSC19.11), 0.5–1×10^6^ human CD34^+^ cord blood cells (AllCells, Emeryville, CA, http://www.allcells.com) were expanded in StemSpan-SFEM (StemCell Technologies, Vancouver, BC, http://www.stemcell.com), supplemented with FTK (100 ng mL^−1^ FLT3L, 10 ng mL^−1^ TPO and 100 ng mL^−1^ KITLG (SCF) (R&D Systems)) in 1 well of a 12-well plate for 3 days. All reprogramming culture steps were conducted in tissue culture plates that were tightly wrapped in Saran wrap. After three days, 0.5×10^6^ cells were nucleofected with 6 µg total plasmid DNA as above using CD34^+^ nucleofector solution (VPA-1003, Lonza) and program U-008. Cells were incubated in RPMI/10% FBS for 4–6 hr then, washed in SFEM and re-plated onto 6 wells of a Retronectin (Takara Bio, Madison, WI, http://www.takara-bio.com)-coated (10 µg mL^−1^) 6-well plate seeded with irradiated (2000 cGy) human mesenchymal stem cells (hMSC, AllCells, seeded at 2×10^4^ cells cm^−2^) in SFEM+FTK. On Day 3, cells were harvested (300 g, 5 min), and re-plated onto irradiated MEF in SFEM+FTK, as above. On Day 4, two mL of hESC medium containing 40 ng mL^−1^ FGF2 was added to CD34^+^/MEF co-cultures. On Day 6, and every 2 days thereafter, half of the medium in each well was removed, cells were collected by centrifugation and media was aspirated and replace with fresh media and returned to the wells. Starting on Day 10, medium was changed every day using MEF conditioned medium supplemented with 40 ng mL^−1^ FGF2. ESC-like colonies were visible under these conditions and emerged as early as 7–21 days post-nucleofection, and manually picked for expansion and further characterization.

### Pluripotency marker and teratoma assays

Whole hiPSC colonies were washed in PBS and fixed cold 3.7% PFA in PBS, washed twice in PBS and permeabilized with 0.2% Triton-X (Sigma) in TBS for 10 min where appropriate. Cells were then incubated with 10% goat or donkey serum (Sigma) in PBS for 1 hr at RT and incubated overnight at 4°C with anti-human NANOG (1∶500, goat IgG1, AF1997, R&D systems,), anti-human POU5F1 (OCT4, 1∶200, mouse IgG1), anti-human SSEA4 (1∶200, mouse IgG1,) (BD Biosciences) anti-human TRA-1-60 (1∶200, mouse IgM, Millipore) diluted in antibody diluent (Invitrogen). Cells were washed 3 times in TBS-T then incubated for 45 min at RT in the dark with secondary antibodies Cy3-conjugated donkey anti-goat IgG1 (1∶1000, Jackson Immuno Research, West Grove, PA), Alexa Fluor 488-conjugated goat anti-mouse IgG1 (1∶500, Invitrogen), or Alexa Fluor 488-conjugated goat anti-mouse IgM (1∶500, Invitrogen), which were diluted in antibody diluent (Invitrogen). Cells were then washed 3 times in TBS-T and mounted with ProLong Gold with DAPI (Invitrogen) onto Superfrost Plus (VWR) slides and imaged with a Confocal Microscope (Zeiss). Teratoma assays were performed as previously described [56].

### Genomic Southern Blotting

5 µg of genomic DNA from hiPSC samples growing in feeder-free conditions was isolated with a DNeasy Blood & Tissue Kit (QIAGEN), and digested with high fidelity restriction enzymes BamHI and SpeI (NEB). Undigested “combination 6” pCEP4 episomal vectors (0.4×: 8.55 pg of pEP4O2DEN2L, 9.72 pg of pEP4EO2SET2K, 9.22 pg of pEP4EO2SEM2K) were mixed in 1∶1∶1 ratios and purified by QIAquick PCR Purification Kit (QIAGEN). Samples were processed using DIG High Prime DNA labeling and Detection Starter Kit II (Roche Applied Biosciences) following the manufacturer's directions. The pCEP4 parental construct (Invitrogen) was digested with NotI and NruI (Invitrogen) to release a 7.3 kb episomal vector backbone probe, gel-extracted using the QIAquick Gel Extraction Kit (Invitrogen), DIG labeled, further digested with AluI (Invitrogen), and purified using QIAquick PCR Purification Kit (QIAGEN).

### Genomic PCR, semi-quantitative RT-PCR, and real-time qRT-PCR

DNA was extracted using DNeasy Blood & Tissue and RNeasy Mini Kits (QIAGEN). For hiPSC experiments, DNA and RNA were extracted from p11 CBiPSC cells, negative control p48 H9 hESC, and positive p2 early CBiPSC, RT was performed using SuperScript-First Strand Synthesis (Invitrogen), and PCR using Pfx DNA polymerase (Invitrogen). Real-time RT-PCR was performed Power SYBR PCR Mastermix (Applied Biosystems). Genomic analysis for non-viral hiPSC used primers as described [25]. RT-PCR analysis used primers as described [55]. For cardiomyocyte analysis, the contents of 16 wells a 96-well plate were removed, RNA extracted as above, cDNA synthesis was performed using a High Capacity RNA-cDNA kit (Applied Biosystems, Carlsbad, CA, http://www.appliedbiosystems.com). Real-time RT-PCR was performed using Universal PCR Master Mix and on an Applied Biosystems 7900HT. Using Taqman Assay-on-Demand Gene Expression Assays (Applied Biosystems) (Table S1).

### Cardiac differentiation

For forced aggregation hEB differentiations, confluent hESC or hiPSC which had been grown on Geltrex as monolayers for 3 to 13 passages were passaged with TrypLE Select and seeded at 2.5×10^6^ per T25 flask. After 24 h growth, cells were treated with TrypLE Select and seeded at 5000 cells per well in 96-well V-bottom uncoated plates (249952, NUNC Rochester, NY, http://www.nuncbrand.com) in 100 µL per well RPMI+PVA medium consisting of RPMI Media 1640 (with L-Glutamine), 4 mg mL^−1^ polyvinyl alcohol (P8136 Sigma-Aldrich St. Louis MO, http://www.sigmaaldrich.com), dissolved in RPMI at 4°C for at least 72 h, mixing by inversion every day, 1% chemically defined lipid concentrate, 10 µg mL^−1^ recombinant human insulin (I9278, Sigma-Aldrich), 400 µM 1-thioglycerol (Sigma-Aldrich), 25 ng mL^−1^ human BMP4 and 5 ng mL^−1^ human FGF2 (both from R&D systems), 1 µM Y-27632 (Stemgent, Cambridge, MA, http://www.stemgent.com). This medium is not stable and was made fresh for each experiment. After 48 hours medium was aspirated with a Costar 8-channel aspirator (Corning Life Sciences, Corning, NY, http://www.corning.com) and replaced with RPMI+FBS medium consisting of RPMI Media 1640, 20% FBS (Characterized, Hyclone), 400 µM 1-thioglycerol. On day 4 media was aspirated and replaced with RPMI+INS consisting of RPMI, 1% chemically defined lipid concentrate, 10 µg mL^−1^ recombinant human insulin, 400 µM 1-thioglycerol and hEB were transferred to 96-well U-bottom tissue culture treated plates (NUNC). Media was changed on d7 and every 3 days afterwards. hEB were visually assessed for contraction on d9 using a Nikon Eclipse Ti microscope (Nikon Instruments, Melvin, NY, http://www.nikoninstruments.com). Images were captured using NIS-Elements (Nikon). Other factors that were tested include: Germcell human serum, Benchmark FBS (Gemini, Sacramento, CA, http://www.gembio.com), Growth factor reduced Matrigel (BD Biosciences), NODAL, activin A, DKK1, VEGFA_165_, WNT3A, TDGF1, BMP2, BMP6, TGFB, IGF1, IGF2, Nidogen (R&D systems), 96-well U-bottom uncoated plates, 96-well F-bottom tissue culture plates (NUNC), ITS-X, ITS-G, N2 supplement, B27 supplement, non-essential amino acids, DMEM, IMDM, F12, KO-DMEM, StemPro-34, KnockOut Serum Replacement, Xeno-free Knockout Serum Replacement, Qualified FBS (all from Invitrogen), X-VIVO 10 (Lonza), BSA (A3311), human serum albumin (HSA), recombinant human albumin (rHA), human transferrin, L-ascorbic acid, L-ascorbic acid-2-phosphate, Stemline II (Sigma-Aldrich), mTeSR1, SFEM, ES-Cult FBS for Hematopoietic Differentiation (StemCell Technologies), mouse WNT3A, EX-CYTE (Millipore, Billerica, MA, http://www.millipore.com). Traditional cardiac differentiations using FBS were performed as previously described [6]. Briefly, confluent H9 hESC grown as colonies on MEF were treated with collagenase IV for 5 min at 37°C then washed from the plate using a 5 mL pipette. Cell clusters were then transferred to Petri dishes in DMEM (with Glutamine), 20% FBS (Characterized, Hyclone), 1% NEAA, 100 µM 2-mercaptethanol medium for 7 days. hEB were then transferred to gelatin coated tissue culture plates. Media was changed every three days.

### Statistical Design

One single replicate consists of one 96-well plate. Repeat replicates were performed 1–4 months apart. Each experiment was repeated >3 times representing >288 hEB. Total number of hEB assessed in this work exceeds 80,000. Wells in which no hEB was detected due to pipetting error were excluded and accounted for approximately 1–5% of wells. P-values were established using an unpaired two-tailed Student's t-test.

### Cardiomyocyte immunocytochemistry and flow cytometry analysis

Whole cardiomyocyte hEB clusters were plated onto fibronectin-coated glass coverslips and given 5 days to attach and processed as above with primary antibodies anti human sarcomeric alpha actinin (1∶200, monoclonal mouse IgG1, ab9465, Abcam, Cambridge, MA, http://www.abcam.com) and anti-human cardiac troponin I (1∶200, monoclonal mouse IgG2b, T8665-13F, US Biological, Swamscott, MA, http://www.usbio.net) and secondary antibodies Alexa Fluor 568 Goat anti-mouse IgG (1∶200, Invitrogen) and Alexa Fluor 488 Goat anti-mouse IgG2b (1∶200, Invitrogen). For flow cytometry, one whole plate of d9 H9 hEB (whole well contents) were disaggregated using TrypLE and stained as above. Cells were analyzed using a FACSCaliber (BD Biosciences) flow cytometer (Beckton-Dickinson). Data was analyzed using FlowJo (Tree Star, Ashland, OR, http://www.treestar.com).

### Cardiomyocyte electrophysiology

For optical micromapping, contracting hEB were mechanically dissected, plated on fibronectin (BD Biosciences)-coated glass coverslips and given at least 5 days to attach. hEB were then stained with either 10 µM Rhod-2-AM calcium dye (Invitrogen) for 20 minutes or 10 µM di-4-ANEPPS voltage dye (Invitrogen) for 5 minutes. After several rinses with Tyrode's solution (135 mM NaCl, 5.4 mM KCl, 1.8 mM CaCl_2_, 1 mM MgCl_2_, 0.33 mM NaH_2_PO_4_, 5 mM HEPES, and 5 mM glucose, (Sigma)), hEB were incubated with 30 µM blebbistatin (Sigma) for 15 minutes to inhibit excitation-contraction coupling and subsequently prevent signal distortion due to motion artifact. The absence of hEB contraction was confirmed visually. hEB were then excited at 530 nm to visualize spontaneous activity and response to electrical field stimulation. Imaging of transmembrane potential (V_m_) or intracellular calcium (Ca_i_) was performed using an Andor iXon+ 860 (Andor Technology, South Windsor, CN, http://www.andor.com) electron multiplying charged coupled device (EMCCD) camera (128×128 pixels) at 490 Hz sampling rate. At 6× magnification, the field of view is ∼520 µm×520 µm, resulting in a spatial resolution of ∼4 µm. Micromapping experiments were performed at room temperature. Macromapping of hESC-CM monolayers was performed using contact fluorescent imaging, in which maps of V_m_ were recorded by placing the monolayer directly on top of a bundle of 253 optical fibers 1 mm in diameter, arranged in a tightly packed, 17-mm-diameter hexagonal array. The cell monolayers were stained with 10 µM di-4-ANEPPS, and continually superfused with Tyrode's solution. The monolayer was excited by an array of high-power green LEDs placed directly above the experimental chamber. The fluorescent dye signal was relayed by the optical fiber bundle to an array of photodetectors and amplifiers, digitized at a 1 kHz sampling rate, and processed by custom written software. Macromapping experiments were performed at 36°C. In drug-response experiments, drugs were added for 15 min before subsequent recordings. To analyze data, the individual recorded signals were spatially filtered using a 5×5 box filter, temporally filtered using a 10 point median filter, baseline-corrected by subtraction of a fitted 3^rd^ order polynomial, and range-normalized. The activation time at each recording site was computed as the time of the maximum first derivative of the action potential (dV_m_/dt_max_) or calcium transient upstroke (dCa/dt_max_). Repolarization time was computed as the 80% recovery time from the peak amplitude, and action potential duration (APD) was computed from the difference of repolarization and activation times. APD maps were computed by first spatially binning voltage data to 16×16 pixels and measuring APD at each pixel. Uniformity of APD was assessed by the coefficient of variation. For each hEB, the coefficient of variation was determined from the mean APD (over all pixels in the APD map), divided by the standard deviation. Conduction velocity was computed by taking the distance of a path perpendicular to the direction of propagation, and dividing by the difference of activation times at the path endpoints. At least 3 paths were chosen for each measurement. The conduction velocity coefficient of variation was determined from the mean conduction velocity (over all measured paths), divided by the standard deviation.

## Supporting Information

Figure S1 Schematic representation of the variables considered whilst optimizing the cardiac differentiation system.
(TIF)

Figure S2 Development of a strategy for the optimization of cardiac differentiation. (**A**) Schematic of previous cardiac differentiation strategy published in Burridge *et al.*, 2007. (**B**) Pilot experiments allowed us to develop a prototype system in which we used BMP4 from d0–d4 and removed the mass culture step to eliminate the inter-hEB paracrine effect and prevents hEB from adhering to each other. (**C**) Final four step optimized differentiation strategy detailing the use of hESC/hIPSC passaged one day prior to aggregation, 5,000 cells in RPMI+PVA media for 2 days followed by 2 days in RPMI+FBS and finally adherence in RPMI+INS. All variables previously tested between the prototype system stage and final system were repeated three times to confirm dose-response under final system conditions.
(TIF)

Figure S3 Optimizing media formulations of hEB formation. (**A**) 21 existing media formulations were compared for efficiency of hEB formation both from cells cultured as colonies on MEF and cells grown as monolayers. hEB were formed from 10,000 cells, collected into a single well on d2 and imaged (4× magnification). The results demonstrated that media formulation from Wiles & Johansson '98 was most successful for homogeneous hEB formation. (**B**) The minimal media requirements for hEB formation were assessed by subtraction until we found that only the combination of a basal medium supplemented with 1 mg mL^−1^ PVA and insulin was required for successful hEB formation (4× magnification). Details of references are provided in References S1.
(TIF)

Figure S4 Optimization of day 0–2 media formulation and growth factor variables using H9 hESC. d9 was chosen for assessment as ‘prototype’ version of our previous protocol had identified this as the day of maximum percentage contraction. Optimal conditions for the d0–d2 phase 2 stage were derived using RPMI-PVA as the base media (Table 1) and making relevant subtractions or additions to it. Optimal conditions for high efficiency differentiation differentiation were: (**A**) 25 ng mL^−1^ of BMP4. (**B**) 5 ng ml^−1^ FGF2. (**C**) The basal medium RPMI 1640. (**D**) No additional L-glutamine other than that included in the basal medium (2.5 mM). (**E**) 400 µM 1-thioglycerol was optimal for cardiac differentiation whereas 2-mercaptoethanol was not suitable for hEB formation. (**F**) 10 µg mL^−1^ insulin, more complex products such as ITS-G or -X (Invitrogen) provided similar results. (**G**) The addition of transferrin did not affect differentiation. (**H**) L-ascorbic acid had a negative dose-response on differentiation. (**I**) 1× chemically defined lipids. (**J**) non-essential amino acids did not enhance differentiation. (**K**) The addition of BSA alone did not promote differentiation although PVA was successful at a concentration of >1 mg mL^−1^. (**L**) Only a comparatively low dose of 1 µM of Y-27632 (ROCK inhibitor) promoted efficient subsequent differentiation. *n* = 3. Error bars, ±S.E.M.
(TIF)

Figure S5 Optimization of forced aggregation hEB formation physical factors. Optimal conditions for physical factors stage were derived using system described in Figure 1A and making relevant subtractions or additions to it. (**A**) Forced aggregation input cell number per well between 500–20,000 cells. 3,000–10,000 cells were suitable for successful cardiac differentiation. hEB did not form from 500 or 1,000 cells. (**B**) Both V-bottom and U-bottom plates were successful for hEB formation, V-bottom plates were chosen due to the comparative ease of media change and prevention of loss of hEB. (**C**) Only day 2 was suitable for change of media RPMI-FBS. (**D**) hEB that were transferred to adherent plates before d4 quickly lost their structure. (**E**) U-bottom plates were chosen over F-bottom plates as hEB would adhere in the center of the well simplifying the observation of contracting hEB. (**F**) Once Y-27632 was added to the media it was found that g-force was no longer required to induce aggregation. (**G**) The density at which the T25 flasks of pluripotent cells were split to the day before forced aggregation did not affect subsequent differentiation. (**H**) Passaging cells one day prior to forced aggregation rather than allowing them to grow to confluence was found to be crucial for efficient differentiation. *n* = 3. Error bars, ±S.E.M.
(TIF)

Figure S6 Optimization of day 2–4 media factors. (**A**) Only 20% fetal bovine serum (FBS) was suitable for inducing >90% contracting hEB. Optimal conditions for the d2–d4 phase 3 stage were derived using RPMI-FBS as the base media (Table 1) and making relevant subtractions or additions to it. (**B**) Manufacturer of FBS did not affect cardiac induction. FBS could be substituted with 20% human serum with no reduction in efficiency to create a fully xeno-free system. The use of Knockout Serum Replacement (KSR) or Xeno-Free KSR was not sufficient to allow cardiomyocyte induction. (**C**) The addition of BMP4 at this d2–d4 stage did not enhance cardiac differentiation. (**D**) The addition of FGF2 also did not have an effect on cardiac differentiation. (**E**) As with d0–d2, only the basal medium RPMI was suitable for efficient cardiac differentiation. (**F**) Additional L-glutamine did not enhance cardiac differentiation. (**G**) 1-thioglycerol was the most suitable thiol for this phase. (**H**) Any level of supplementation with insulin during this phase completely ablated cardiac differentiation. (**I**) Human transferrin did not enhance differentiation. (**J**) L-ascorbic acid did not enhance differentiation at low dose although did have a negative effect on differentiation at high doses (560 µM). (**K**) Chemically defined lipids dis not enhance differentiation although EX-CYTE (Millipore) had a negative effect. (**L**) Non-essential amino acids (NEAA) did not enhanced this phase of differentiation. *n* = 3. Error bars, ±S.E.M.
(TIF)

Figure S7 Optimization of day 4 onwards media formulation. Optimal conditions for the d0–d2 phase 2 stage were derived using RPMI-INS as the base media (Table 1) and making relevant subtractions or additions to it. (**A**) FBS, PVA or HSA was not required for the d4 onwards phase. (**B**) In contrast to the d2–d4 stage, insulin did not effect this d4+ phase. (**C**) Transferrin was not required. (**D**) Supplemental lipids were also not required. (**E**) 1-thioglycerol was essential for this d4+ phase. Although only RPMI+1-thioglycerol was required for d4+ cardiac differentiation, a more complex media (RPMI-INS) was required for further (d9 onwards) hEB survival and therefore used in the final system. *n* = 3. Error bars, ±S.E.M.
(TIF)

Figure S8 Demonstration of cardiomyocyte drug responsiveness and electrical coupling using optical mapping. (**A**) Time series of voltage maps demonstrates electrical coupling in an hESC-derived cardiomyocyte monolayer during 0.67 Hz pacing (pulse symbol indicates stimulus site, arrows indicate direction of propagation). A second, spontaneous activation site can seen on the upper right at 40 ms. b, Representative V_m_ traces, time aligned by the stimulus timing, taken at site x in a. Isoproterenol and pinacidil shortened the action potential (363±137 ms control (*n* = 73 recording sites) vs. 257±56 ms pinacidil (*n* = 64) vs. 262±107 ms isoproterenol (*n* = 94), mean±s.d.). (**B**) Electrical coupling between two hEB during voltage micromapping. a, Phase map of two hEB in close contact at 6×. b, Time series of voltage maps demonstrates electrical coupling between the hEB pair by continuous propagation from one hEB to the other. c and d, V_m_ traces (from the three boxes in a) demonstrate the synchrony of the action potentials, as the electrical wave propagates from right to left (red to blue to green trace) across the field of view.
(TIF)

Figure S9 Optimization of xeno- and serum-free day 2–4 media formulation. Optimal conditions for the d2–d4 phase 2 stage were derived using ‘Xeno-free’ as the base media (Table 1) and making relevant subtractions or additions to it. (**A**) PVA supplementation did not induce cardiac differentiation. (**B**) A high dose (5 mg mL^−1^) of human serum albumin (HSA) was required. (**C**) HSA could not be replaced by recombinant albumin. (**D**) As with the xeno-containing d2–d4 media formulation, the addition of insulin inhibited cardiac differentiation. (**E**) The addition of transferrin did not impact differentiation. (**F**) L-ascorbic acid promoted xeno-free cardiac differentiation any concentration. (**G**) Chemically defined lipids had a small effect. (**H**) Non-essential amino acids were not required. This xeno-free d2–d4 media could also be simply replaced by the BSA-containing media StemPro34 (Invitrogen) supplemented with 280 µM of L-ascorbic acid with similar results (data not shown).
(TIF)

Table S1 Real-time RT-PCR primers. Table of Applied Biosystems assay-on-demand primers used for real-time RT-PCR analysis.
(TIF)

Table S2 Heat-map of optimized media formulations and physical factors for cardiac differentiation of H9 hESC. A condensed schematic of the optimal cardiac differentiation media formulations and physical factors used in the optimized protocol. Red represents greater than 90% of hEB contracting on d9, yellow represents 50–90% of hEB contracting on d9, blue represents less than 50% of hEB contracting on d9, and white represents 0% contracting hEB on d9.
(TIF)

Movie S1 Contracting H9 hEB on d9 of differentiation.
(MOV)

Movie S2 Contracting iPS(IMR90)-1 hEB on d9 of differentiation.
(MP4)

Movie S3 Voltage mapping of H9 hEB.
(MP4)

Movie S4 Calcium mapping of H9 hEB.
(MP4)

References S1 Supplementary references.
(DOC)
