# Supplementary figures and images for: A Universal System for Highly Efficient Cardiac Differentiation of Human Induced Pluripotent Stem Cells That Eliminates Interline Variability
Source: PLoS One. 2011 Apr 8;6(4):e18293. doi: 10.1371/journal.pone.0018293 (PMC3072973; doi:10.1371/journal.pone.0018293)

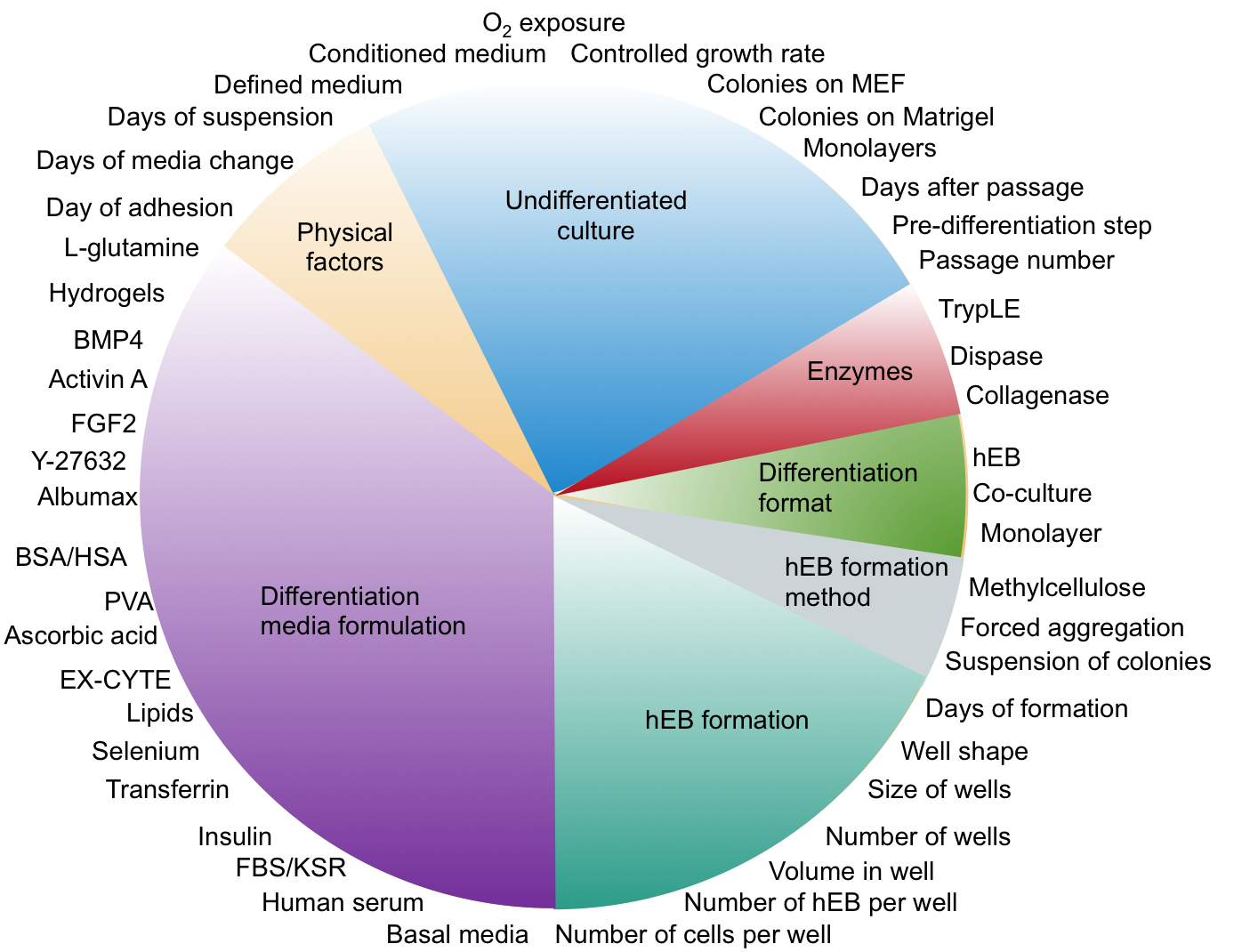

Supplement: Figure S1 — Schematic representation of the variables considered whilst optimizing the cardiac differentiation system. (TIF) [file pone.0018293.s001.tif]

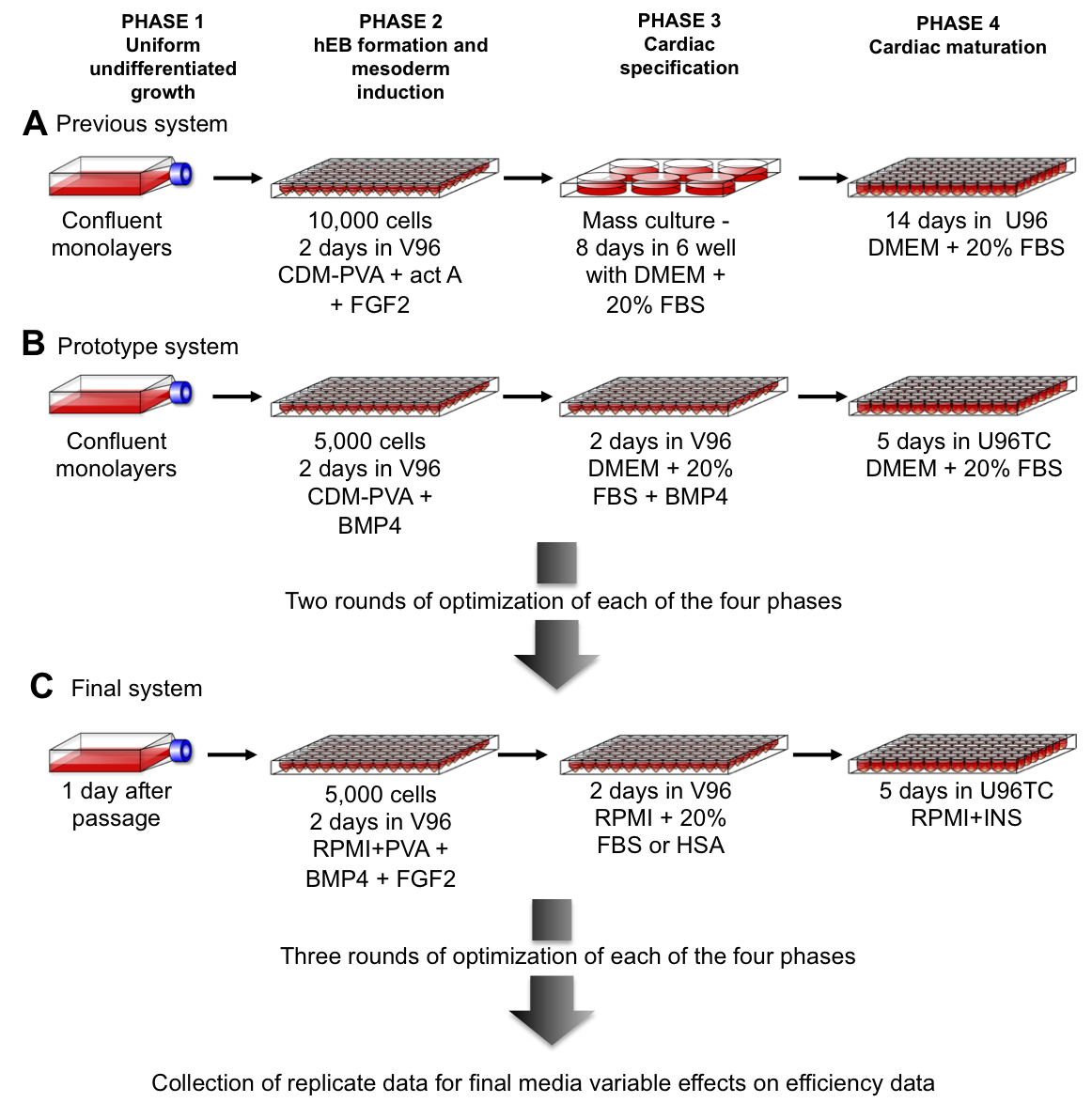

Supplement: Figure S2 — Development of a strategy for the optimization of cardiac differentiation. (A) Schematic of previous cardiac differentiation strategy published in Burridge et al., 2007. (B) Pilot experiments allowed us to develop a prototype system in which we used BMP4 from d0–d4 and removed the mass culture step to eliminate the inter-hEB paracrine effect and prevents hEB from adhering to each other. (C) Final four step optimized differentiation strategy detailing the use of hESC/hIPSC passaged one day prior to aggregation, 5,000 cells in RPMI+PVA media for 2 days followed by 2 days in RPMI+FBS and finally adherence in RPMI+INS. All variables previously tested between the prototype system stage and final system were repeated three times to confirm dose-response under final system conditions. (TIF) [file pone.0018293.s002.tif]

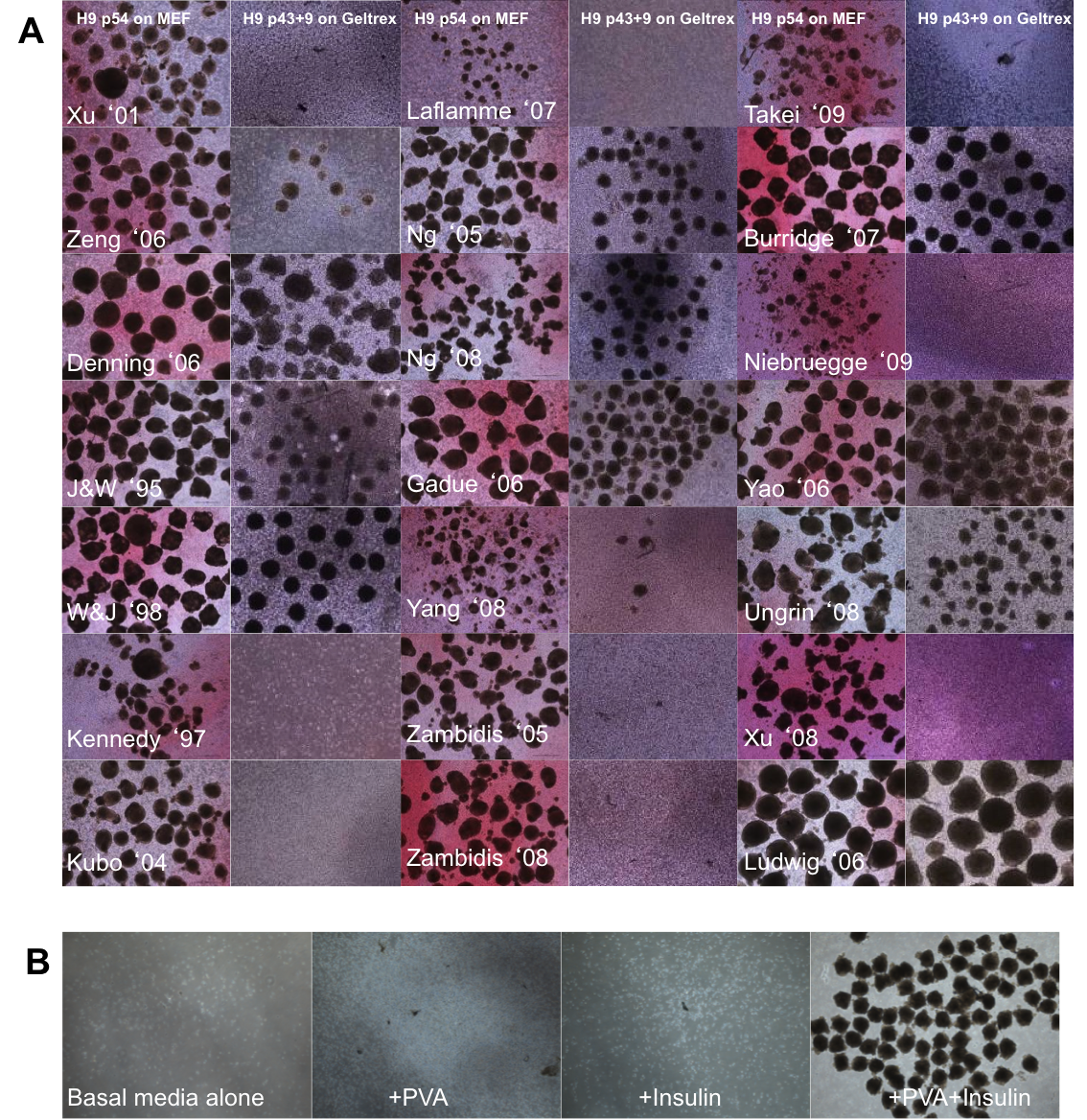

Supplement: Figure S3 — Optimizing media formulations of hEB formation. (A) 21 existing media formulations were compared for efficiency of hEB formation both from cells cultured as colonies on MEF and cells grown as monolayers. hEB were formed from 10,000 cells, collected into a single well on d2 and imaged (4× magnification). The results demonstrated that media formulation from Wiles & Johansson '98 was most successful for homogeneous hEB formation. (B) The minimal media requirements for hEB formation were assessed by subtraction until we found that only the combination of a basal medium supplemented with 1 mg mL−1 PVA and insulin was required for successful hEB formation (4× magnification). Details of references are provided in References S1. (TIF) [file pone.0018293.s003.tif]

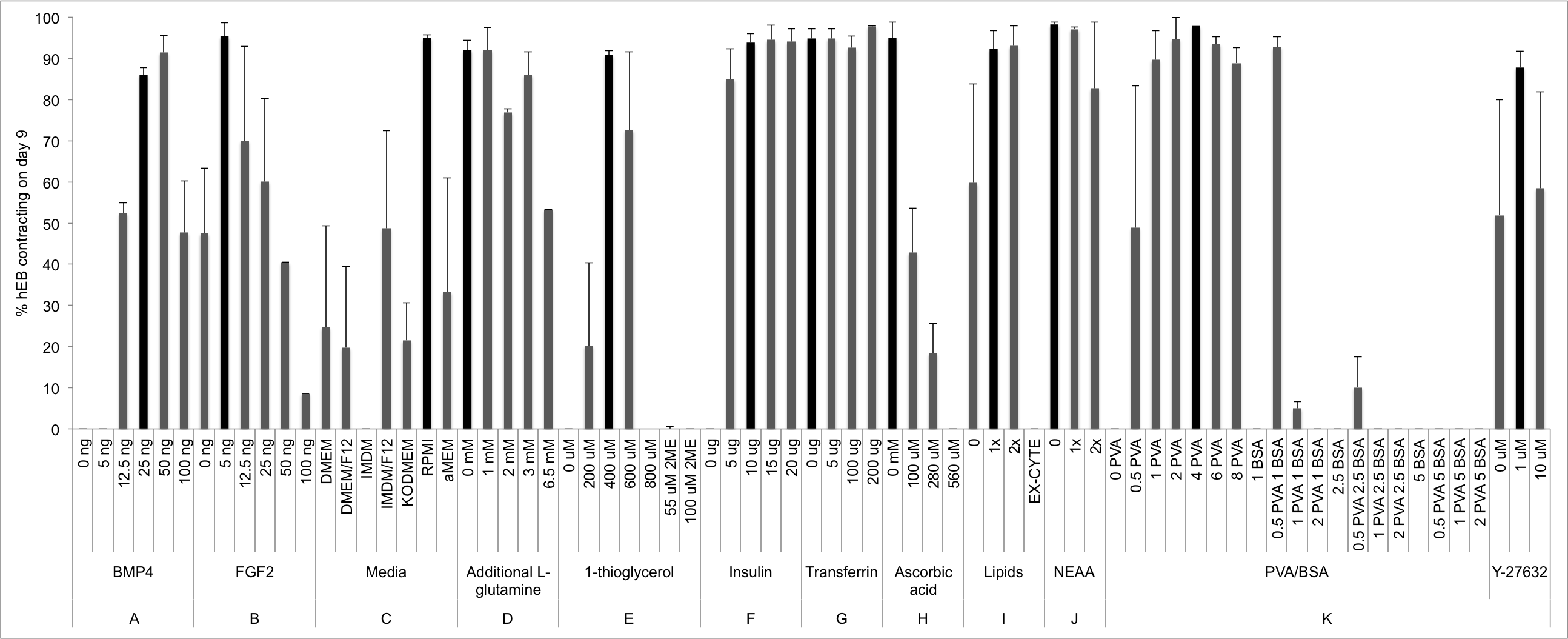

Supplement: Figure S4 — Optimization of day 0–2 media formulation and growth factor variables using H9 hESC. d9 was chosen for assessment as ‘prototype’ version of our previous protocol had identified this as the day of maximum percentage contraction. Optimal conditions for the d0–d2 phase 2 stage were derived using RPMI-PVA as the base media (Table 1) and making relevant subtractions or additions to it. Optimal conditions for high efficiency differentiation differentiation were: (A) 25 ng mL−1 of BMP4. (B) 5 ng ml−1 FGF2. (C) The basal medium RPMI 1640. (D) No additional L-glutamine other than that included in the basal medium (2.5 mM). (E) 400 µM 1-thioglycerol was optimal for cardiac differentiation whereas 2-mercaptoethanol was not suitable for hEB formation. (F) 10 µg mL−1 insulin, more complex products such as ITS-G or -X (Invitrogen) provided similar results. (G) The addition of transferrin did not affect differentiation. (H) L-ascorbic acid had a negative dose-response on differentiation. (I) 1× chemically defined lipids. (J) non-essential amino acids did not enhance differentiation. (K) The addition of BSA alone did not promote differentiation although PVA was successful at a concentration of >1 mg mL−1. (L) Only a comparatively low dose of 1 µM of Y-27632 (ROCK inhibitor) promoted efficient subsequent differentiation. n = 3. Error bars, ±S.E.M. (TIF) [file pone.0018293.s004.tif]

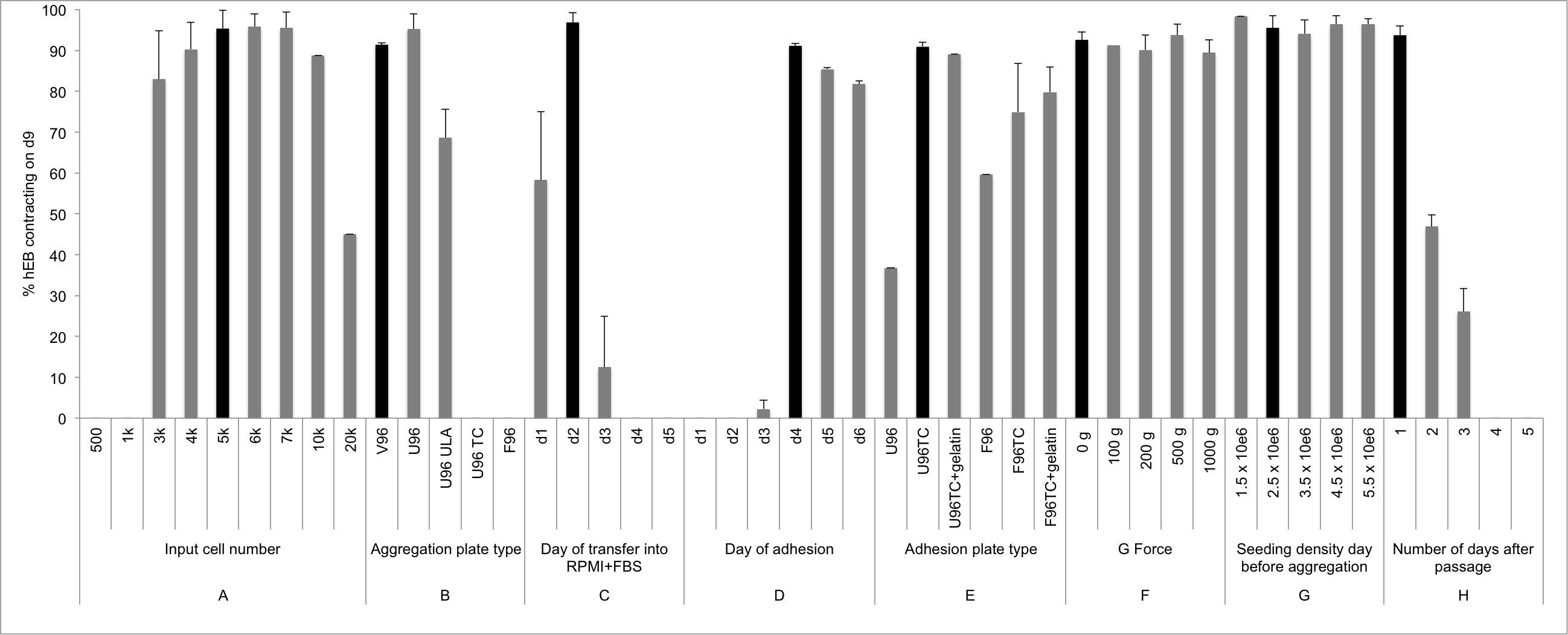

Supplement: Figure S5 — Optimization of forced aggregation hEB formation physical factors. Optimal conditions for physical factors stage were derived using system described in Figure 1A and making relevant subtractions or additions to it. (A) Forced aggregation input cell number per well between 500–20,000 cells. 3,000–10,000 cells were suitable for successful cardiac differentiation. hEB did not form from 500 or 1,000 cells. (B) Both V-bottom and U-bottom plates were successful for hEB formation, V-bottom plates were chosen due to the comparative ease of media change and prevention of loss of hEB. (C) Only day 2 was suitable for change of media RPMI-FBS. (D) hEB that were transferred to adherent plates before d4 quickly lost their structure. (E) U-bottom plates were chosen over F-bottom plates as hEB would adhere in the center of the well simplifying the observation of contracting hEB. (F) Once Y-27632 was added to the media it was found that g-force was no longer required to induce aggregation. (G) The density at which the T25 flasks of pluripotent cells were split to the day before forced aggregation did not affect subsequent differentiation. (H) Passaging cells one day prior to forced aggregation rather than allowing them to grow to confluence was found to be crucial for efficient differentiation. n = 3. Error bars, ±S.E.M. (TIF) [file pone.0018293.s005.tif]

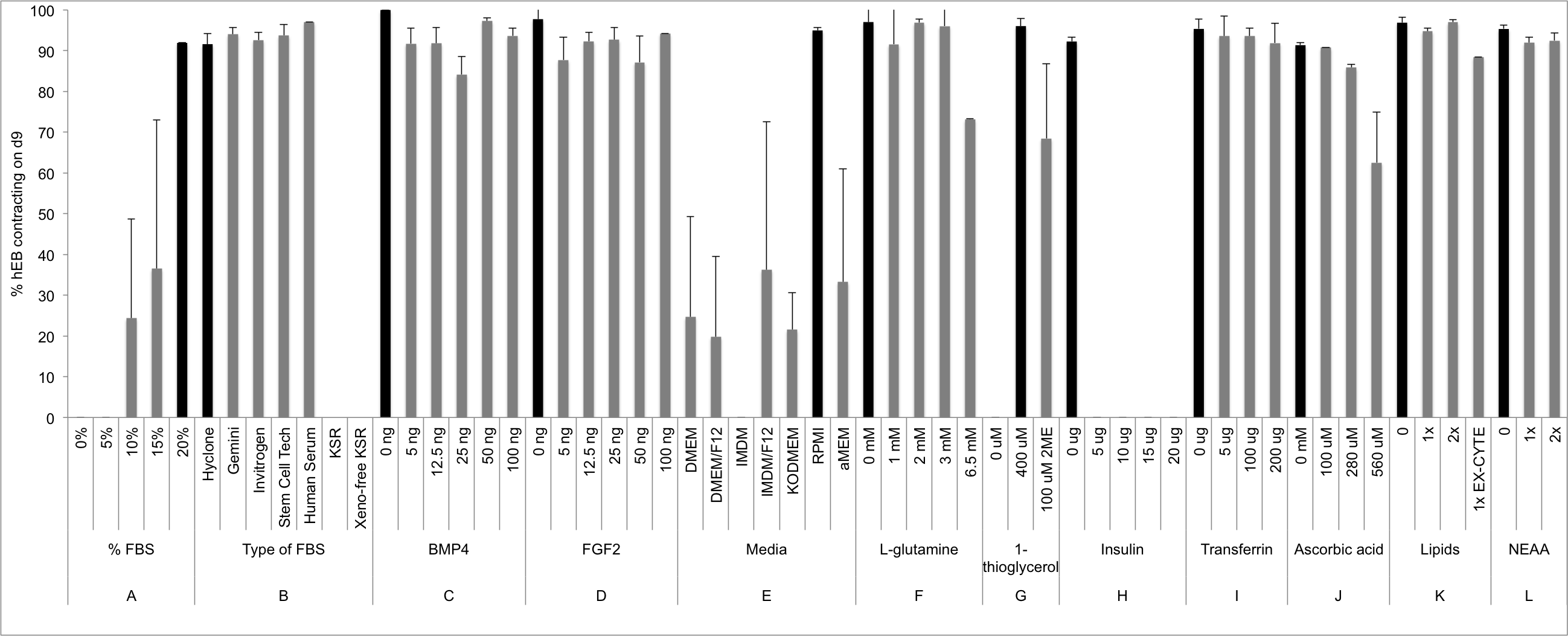

Supplement: Figure S6 — Optimization of day 2–4 media factors. (A) Only 20% fetal bovine serum (FBS) was suitable for inducing >90% contracting hEB. Optimal conditions for the d2–d4 phase 3 stage were derived using RPMI-FBS as the base media (Table 1) and making relevant subtractions or additions to it. (B) Manufacturer of FBS did not affect cardiac induction. FBS could be substituted with 20% human serum with no reduction in efficiency to create a fully xeno-free system. The use of Knockout Serum Replacement (KSR) or Xeno-Free KSR was not sufficient to allow cardiomyocyte induction. (C) The addition of BMP4 at this d2–d4 stage did not enhance cardiac differentiation. (D) The addition of FGF2 also did not have an effect on cardiac differentiation. (E) As with d0–d2, only the basal medium RPMI was suitable for efficient cardiac differentiation. (F) Additional L-glutamine did not enhance cardiac differentiation. (G) 1-thioglycerol was the most suitable thiol for this phase. (H) Any level of supplementation with insulin during this phase completely ablated cardiac differentiation. (I) Human transferrin did not enhance differentiation. (J) L-ascorbic acid did not enhance differentiation at low dose although did have a negative effect on differentiation at high doses (560 µM). (K) Chemically defined lipids dis not enhance differentiation although EX-CYTE (Millipore) had a negative effect. (L) Non-essential amino acids (NEAA) did not enhanced this phase of differentiation. n = 3. Error bars, ±S.E.M. (TIF) [file pone.0018293.s006.tif]

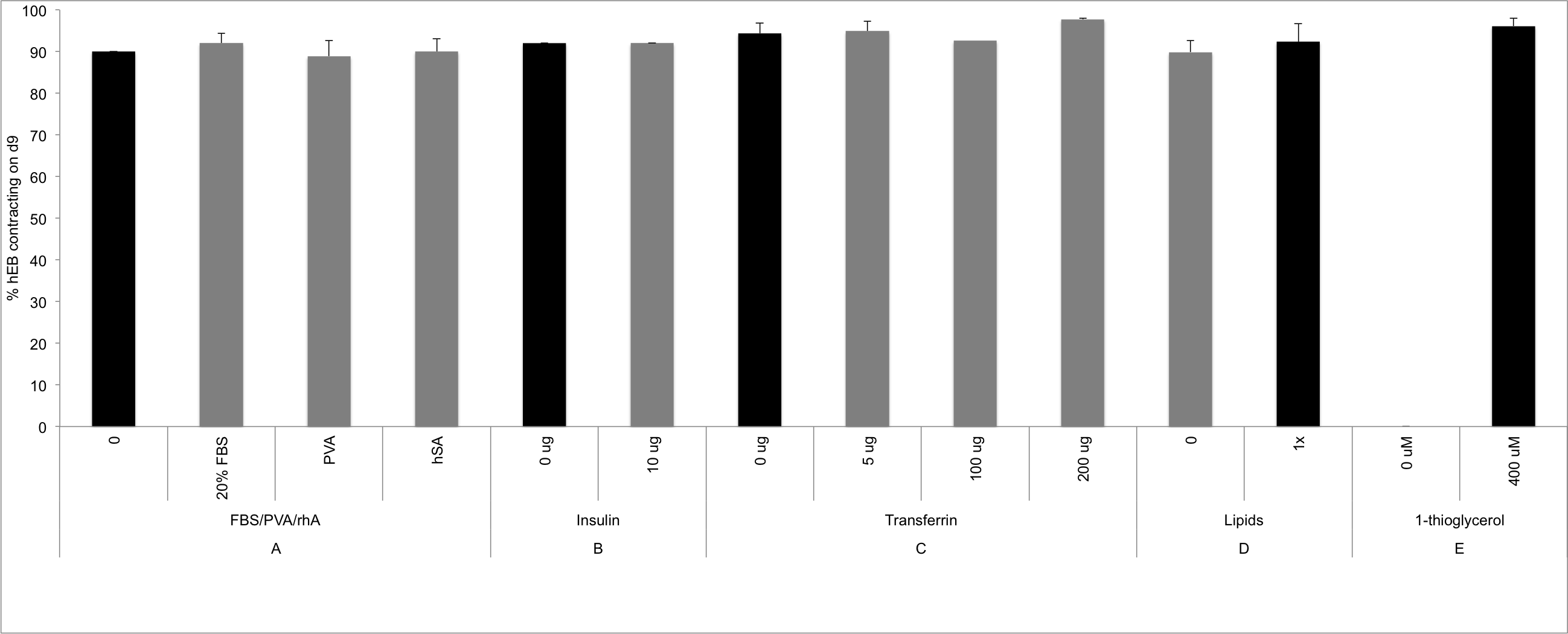

Supplement: Figure S7 — Optimization of day 4 onwards media formulation. Optimal conditions for the d0–d2 phase 2 stage were derived using RPMI-INS as the base media (Table 1) and making relevant subtractions or additions to it. (A) FBS, PVA or HSA was not required for the d4 onwards phase. (B) In contrast to the d2–d4 stage, insulin did not effect this d4+ phase. (C) Transferrin was not required. (D) Supplemental lipids were also not required. (E) 1-thioglycerol was essential for this d4+ phase. Although only RPMI+1-thioglycerol was required for d4+ cardiac differentiation, a more complex media (RPMI-INS) was required for further (d9 onwards) hEB survival and therefore used in the final system. n = 3. Error bars, ±S.E.M. (TIF) [file pone.0018293.s007.tif]

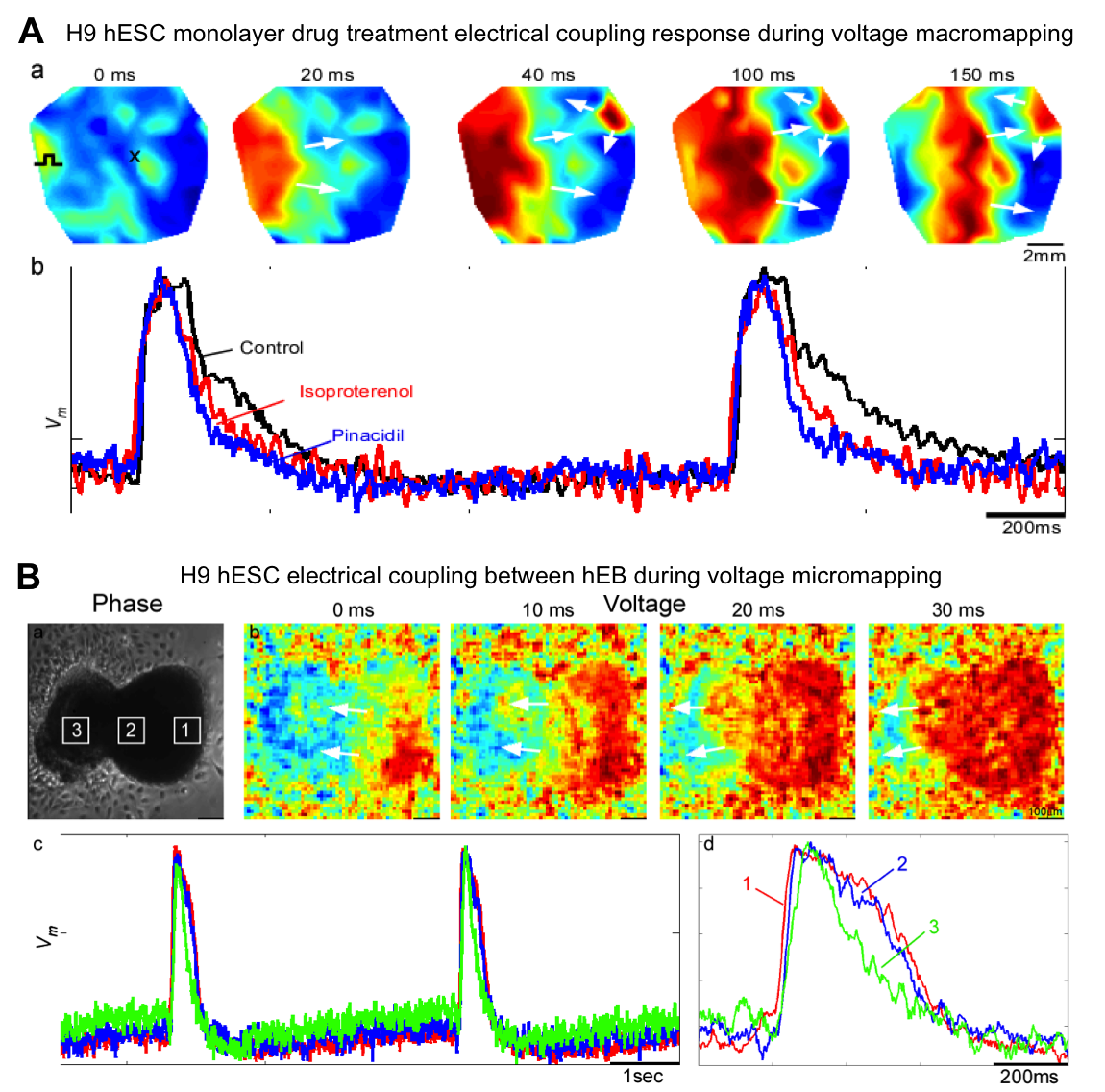

Supplement: Figure S8 — Demonstration of cardiomyocyte drug responsiveness and electrical coupling using optical mapping. (A) Time series of voltage maps demonstrates electrical coupling in an hESC-derived cardiomyocyte monolayer during 0.67 Hz pacing (pulse symbol indicates stimulus site, arrows indicate direction of propagation). A second, spontaneous activation site can seen on the upper right at 40 ms. b, Representative Vm traces, time aligned by the stimulus timing, taken at site x in a. Isoproterenol and pinacidil shortened the action potential (363±137 ms control (n = 73 recording sites) vs. 257±56 ms pinacidil (n = 64) vs. 262±107 ms isoproterenol (n = 94), mean±s.d.). (B) Electrical coupling between two hEB during voltage micromapping. a, Phase map of two hEB in close contact at 6×. b, Time series of voltage maps demonstrates electrical coupling between the hEB pair by continuous propagation from one hEB to the other. c and d, Vm traces (from the three boxes in a) demonstrate the synchrony of the action potentials, as the electrical wave propagates from right to left (red to blue to green trace) across the field of view. (TIF) [file pone.0018293.s008.tif]

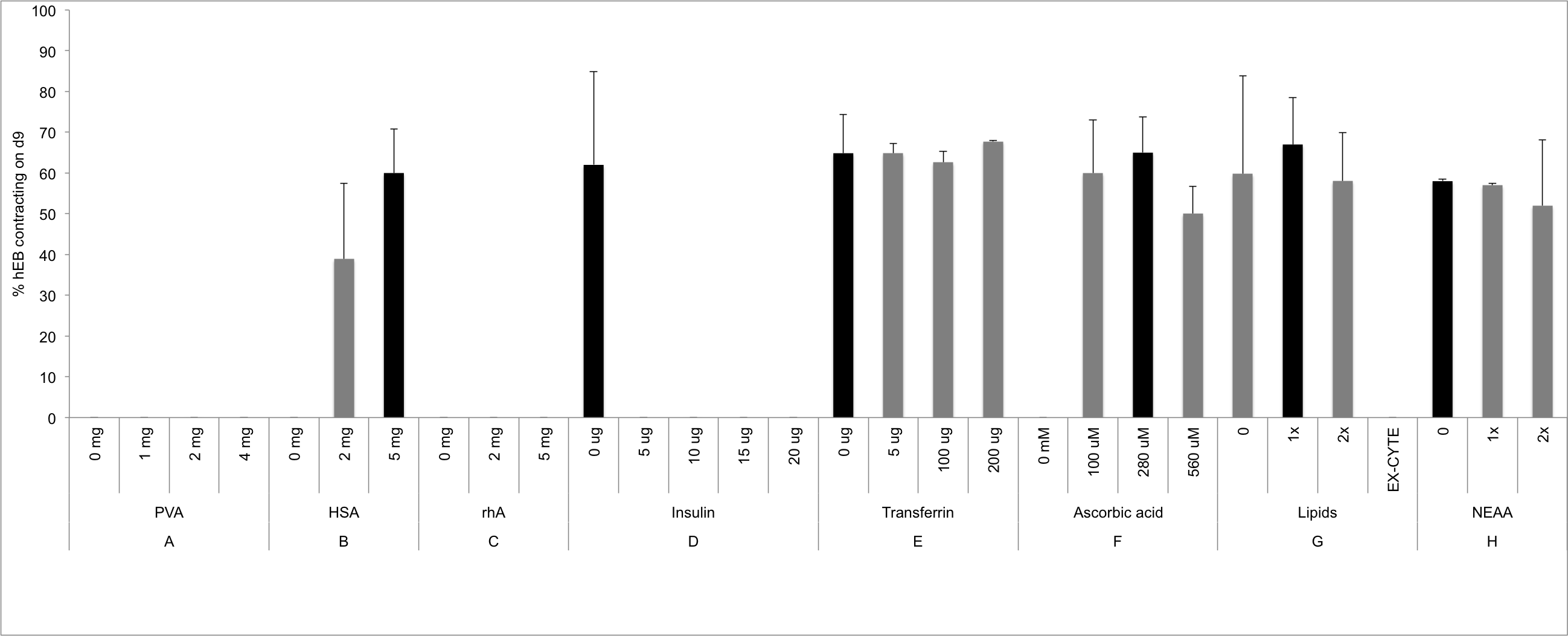

Supplement: Figure S9 — Optimization of xeno- and serum-free day 2–4 media formulation. Optimal conditions for the d2–d4 phase 2 stage were derived using ‘Xeno-free’ as the base media (Table 1) and making relevant subtractions or additions to it. (A) PVA supplementation did not induce cardiac differentiation. (B) A high dose (5 mg mL−1) of human serum albumin (HSA) was required. (C) HSA could not be replaced by recombinant albumin. (D) As with the xeno-containing d2–d4 media formulation, the addition of insulin inhibited cardiac differentiation. (E) The addition of transferrin did not impact differentiation. (F) L-ascorbic acid promoted xeno-free cardiac differentiation any concentration. (G) Chemically defined lipids had a small effect. (H) Non-essential amino acids were not required. This xeno-free d2–d4 media could also be simply replaced by the BSA-containing media StemPro34 (Invitrogen) supplemented with 280 µM of L-ascorbic acid with similar results (data not shown). (TIF) [file pone.0018293.s009.tif]

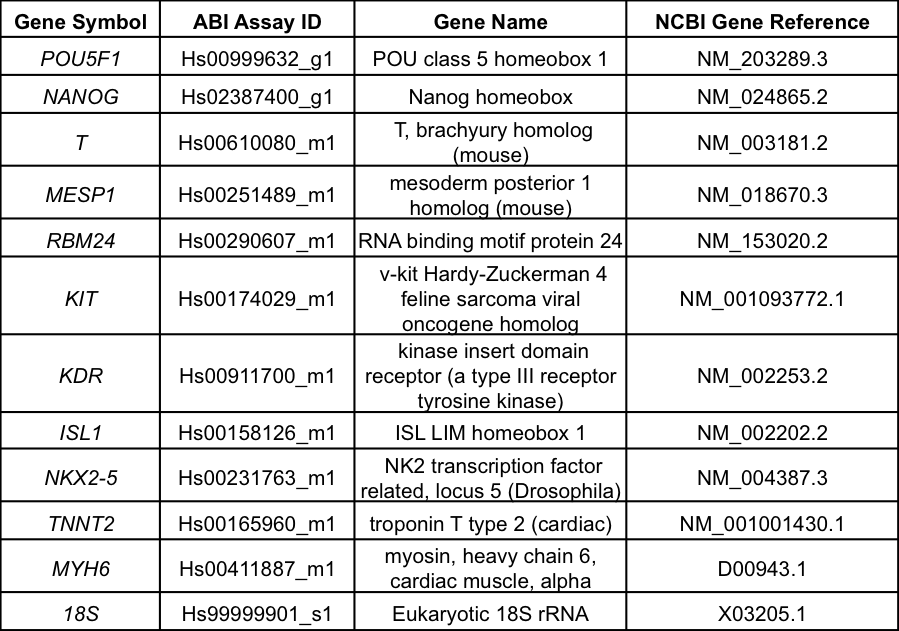

Supplement: Table S1 — Real-time RT-PCR primers. Table of Applied Biosystems assay-on-demand primers used for real-time RT-PCR analysis. (TIF) [file pone.0018293.s010.tif]

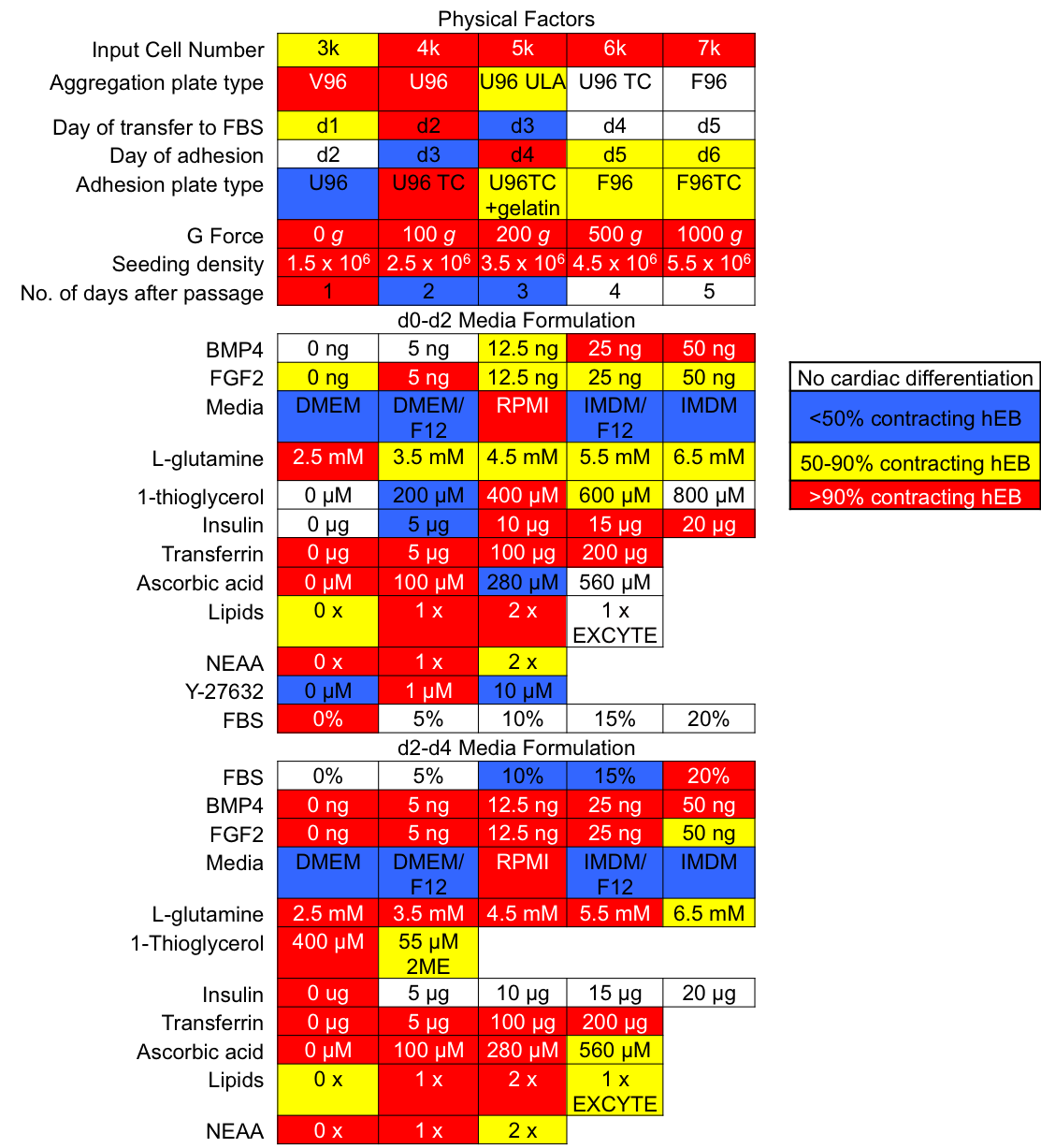

Supplement: Table S2 — Heat-map of optimized media formulations and physical factors for cardiac differentiation of H9 hESC. A condensed schematic of the optimal cardiac differentiation media formulations and physical factors used in the optimized protocol. Red represents greater than 90% of hEB contracting on d9, yellow represents 50–90% of hEB contracting on d9, blue represents less than 50% of hEB contracting on d9, and white represents 0% contracting hEB on d9. (TIF) [file pone.0018293.s011.tif]
