# Supplementary material for: A Universal System for Highly Efficient Cardiac Differentiation of Human Induced Pluripotent Stem Cells That Eliminates Interline Variability
Source: PLoS One. 2011 Apr 8;6(4):e18293. doi: 10.1371/journal.pone.0018293 (PMC3072973; doi:10.1371/journal.pone.0018293)
Supplement: References S1 — Supplementary references. (DOC) [file pone.0018293.s016.doc]

**Supplementary References**

1. Xu C, Inokuma MS, Denham J, Golds K, Kundu P, et al. (2001) Feeder-free growth of undifferentiated human embryonic stem cells. Nat Biotechnol 19: 971-974.

2. Zeng X, Miura T, Luo Y, Bhattacharya B, Condie B, et al. (2004) Properties of pluripotent human embryonic stem cells BG01 and BG02. Stem Cells 22: 292-312.

3. Denning C, Allegrucci C, Priddle H, Barbadillo-Munoz MD, Anderson D, et al. (2006) Common culture conditions for maintenance and cardiomyocyte differentiation of the human embryonic stem cell lines, BG01 and HUES-7. Int J Dev Biol 50: 27-37.

4. Wiles MV, Johansson BM (1999) Embryonic stem cell development in a chemically defined medium. Exp Cell Res 247: 241-248.

5. Kennedy M, Firpo M, Choi K, Wall C, Robertson S, et al. (1997) A common precursor for primitive erythropoiesis and definitive haematopoiesis. Nature 386: 488-493.

6. Kubo A, Shinozaki K, Shannon JM, Kouskoff V, Kennedy M, et al. (2004) Development of definitive endoderm from embryonic stem cells in culture. Development 131: 1651-1662.

7. Laflamme MA, Chen KY, Naumova AV, Muskheli V, Fugate JA, et al. (2007) Cardiomyocytes derived from human embryonic stem cells in pro-survival factors enhance function of infarcted rat hearts. Nat Biotechnol 25: 1015-1024.

8. Ng ES, Davis RP, Azzola L, Stanley EG, Elefanty AG (2005) Forced aggregation of defined numbers of human embryonic stem cells into embryoid bodies fosters robust, reproducible hematopoietic differentiation. Blood 106: 1601-1603.

9. Ng ES, Davis R, Stanley EG, Elefanty AG (2008) A protocol describing the use of a recombinant protein-based, animal product-free medium (APEL) for human embryonic stem cell differentiation as spin embryoid bodies. Nat Protoc 3: 768-776.

10. Gadue P, Huber TL, Paddison PJ, Keller GM (2006) Wnt and TGF-beta signaling are required for the induction of an in vitro model of primitive streak formation using embryonic stem cells. Proc Natl Acad Sci U S A 103: 16806-16811.

11. Yang L, Soonpaa MH, Adler ED, Roepke TK, Kattman SJ, et al. (2008) Human cardiovascular progenitor cells develop from a KDR+ embryonic-stem-cell-derived population. Nature 453: 524-528.

12. Zambidis ET, Peault B, Park TS, Bunz F, Civin CI (2005) Hematopoietic differentiation of human embryonic stem cells progresses through sequential hematoendothelial, primitive, and definitive stages resembling human yolk sac development. Blood 106: 860-870.

13. Zambidis ET, Park TS, Yu W, Tam A, Levine M, et al. (2008) Expression of angiotensin-converting enzyme (CD143) identifies and regulates primitive hemangioblasts derived from human pluripotent stem cells. Blood 112: 3601-3614.

14. Takei S, Ichikawa H, Johkura K, Mogi A, No H, et al. (2009) Bone morphogenetic protein-4 promotes induction of cardiomyocytes from human embryonic stem cells in serum-based embryoid body development. Am J Physiol Heart Circ Physiol 296: H1793-1803.

15. Burridge PW, Anderson D, Priddle H, Barbadillo Munoz MD, Chamberlain S, et al. (2007) Improved human embryonic stem cell embryoid body homogeneity and cardiomyocyte differentiation from a novel V-96 plate aggregation system highlights interline variability. Stem Cells 25: 929-938.

16. Niebruegge S, Bauwens CL, Peerani R, Thavandiran N, Masse S, et al. (2009) Generation of human embryonic stem cell-derived mesoderm and cardiac cells using size-specified aggregates in an oxygen-controlled bioreactor. Biotechnol Bioeng 102: 493-507.

17. Yao S, Chen S, Clark J, Hao E, Beattie GM, et al. (2006) Long-term self-renewal and directed differentiation of human embryonic stem cells in chemically defined conditions. Proc Natl Acad Sci U S A 103: 6907-6912.

18. Ungrin MD, Joshi C, Nica A, Bauwens C, Zandstra PW (2008) Reproducible, ultra high-throughput formation of multicellular organization from single cell suspension-derived human embryonic stem cell aggregates. PLoS ONE 3: e1565.

19. Xu XQ, Graichen R, Soo SY, Balakrishnan T, Rahmat SN, et al. (2008) Chemically defined medium supporting cardiomyocyte differentiation of human embryonic stem cells. Differentiation 76: 958-970.

20. Ludwig TE, Bergendahl V, Levenstein ME, Yu J, Probasco MD, et al. (2006) Feeder-independent culture of human embryonic stem cells. Nat Methods 3: 637-646.
